# Cubebin Attenuates Methamphetamine-Induced Neurotoxicity Through CREB/BDNF/Caspase-3 Signaling: In Vivo and In Silico Study

**DOI:** 10.3390/medicina61091567

**Published:** 2025-08-31

**Authors:** Sattam Khulaif Alenezi, Khalid Saad Alharbi, Tariq G. Alsahli, Muhammad Afzal, Reem ALQahtani, Samiyah Alshehri, Imran Kazmi, Nadeem Sayyed

**Affiliations:** 1 Department of Pharmacology and Toxicology, College of Pharmacy, Qassim University, Buraydah 51452, Al Qassim, Saudi Arabia; sk.alenezi@qu.edu.sa (S.K.A.); khalid.alharbi9@qu.edu.sa (K.S.A.); 2 Department of Pharmacology, College of Pharmacy, Jouf University, Sakaka 72341, Aljouf, Saudi Arabia; tgalsahli@ju.edu.sa; 3 Department of Pharmaceutical Sciences, Pharmacy Program, Batterjee Medical College, Jeddah 21442, Mecca, Saudi Arabia; 4 School of Pharmaceutical Sciences, Swami Rama Himalyan University, Swami Rama Nagar, Jolly Grant, Dehradun 248016, Uttarakhand, India; 5 Department of Pharmaceutical Science, College of Pharmacy, Princess Nourah Bint Abdul Rahman University, Riyadh 11564, Saudi Arabia; rgalqahtani@pnu.edu.sa; 6 Department of Pharmacology and Toxicology, College of Pharmacy, King Saud University, P.O. Box 145111, Riyadh 11452, Saudi Arabia; saalshehri@ksu.edu.sa; 7 Department of Biochemistry, Faculty of Science, King Abdulaziz University, Jeddah 21589, Mecca, Saudi Arabia; ikazmi@kau.edu.sa; 8 Dr. R.G. Bhoyar Institute of Pharmaceutical Education & Research, Wardha 442001, Maharashtra, India; snadeem.pharma@gmail.com

**Keywords:** apoptosis, in silico, methamphetamine, neurotransmitters, neurotoxicity, oxidative stress

## Abstract

*Background and Objectives:* Methamphetamine (METH) is a potent psychostimulant known to induce neurotoxicity and neurodegeneration, leading to cognitive impairment. This study aimed to explore cubebin’s potential neuroprotective effects against METH-induced cognitive deficits by investigating its ability to suppress lipid peroxidation and pro-inflammatory markers and modulate neurotransmitter levels. *Material and Methods:* A total of 30 rats were taken and randomly grouped into five groups: group I—control; group II—METH 100 mg/kg/i.p.; group III—METH + cubebin (10 mg/kg/p.o.); group IV—METH + cubebin (20 mg/kg/p.o.); and group V—cubebin per os at 20 mg/kg. After a 14-day oral regimen, behavioral activities were assessed utilizing the Morris water maze (MWM). Biochemical analysis included neurotransmitters, including dopamine (DA), norepinephrine (NE), and gamma-aminobutyric acid (GABA); oxidative stress markers (malondialdehyde (MDA); nitric oxide (NO), catalase (CAT), reduced glutathione (GSH)); inflammatory cytokines [interleukin (IL-1β), IL-6, tumor necrosis factor-α (TNF-α), nuclear factor kappa-light-chain-enhancer of activated B cells (NF-κB)]; neurotrophic factors (BDNF, CREB); and apoptotic markers (caspase-3 and caspase-9). Furthermore, molecular docking and simulation studies were conducted. *Results:* Treatment with cubebin led to a marked reduction in latency during the MWM task. It significantly modulated the oxidative stress markers (SOD, GSH, CAT, MDA, and NO), inflammatory cytokines (IL-6, IL-1β, TNF-α), neurotrophic factors (CREB, BDNF), apoptotic markers (NFkB, caspase-3, caspase-9), and neurotransmitters (NE, DA, and GABA) in METH-induced memory-impaired rats. The results of molecular dynamics simulation (MDS) provided insight into the mechanisms that associate proteins CREB, BDNF, and caspase-3 in conformational dynamics upon binding to cubebin. *Conclusions:* In conclusion, cubebin administration improved cognitive function in rats by modulating antioxidant enzyme activity, reducing pro-inflammatory cytokines, and regulating neurotransmitter levels, demonstrating its potential neuroprotective effects against MA-induced neurodegeneration.

## 1. Introduction

Neurotoxicity arises from the direct or indirect effects of toxic materials that interrupt the functional activity of the nervous system in humans and animals. A wide array of chemicals can induce neurotoxic ailments in humans. In contrast, others serve as experimental models for assessing physiological and pathological function in vivo, in vitro, and in human cell lines. Several chemicals exert their effects directly on neural cells, while others act on metabolic processes that are crucial for the function of the nervous system [1]. Recent evidence suggests that neurotoxicity may not be solely confined to the nervous system. Systemic effects, particularly on the endocrine, immune, and gastrointestinal systems, may indirectly impact neurological function. Neurotoxic agents may interfere with neural signaling or influence developmental processes that affect the organization and functioning of the mature nervous system. The characteristics of these effects can be acute or subacute, with the development of clinical manifestations ranging from rapid to gradual and with the potential for chronic change. These effects are commonly reversible with the discontinuance of the neurotoxin; the effects may become manifest only after a longer time, or perhaps may remain as long-term neurological deficits in cases of chronic or gross exposure [2].

Methamphetamine (METH) is known to be one of the most common psychostimulant substances, and its abuse poses a significant challenge to the public health sector. Chronic use of METH is associated with different severe neuropsychiatric conditions such as agitation, anxiety, hallucinations, paranoia, and psychosis. Further, several neuropsychiatric deficits belonging to attention, memory, and executive function are characteristic of METH [3].

The neurotoxic effects of METH play a part in these negative outcomes. They also suggest that the drug causes excessive levels of neurotransmitter release through the depletion of synaptic vesicles, blocking the reuptake of neurotransmitters, and suppression of the transporter proteins. The consequent oxidative stress implication with augmented reactive oxygen species (ROS) has been described as central to METH-induced neurotoxicity. Based on these effects, microglial activation and consequent neuroinflammation aggravate these impacts [3,4].

To address the neurotoxic effects of METH, researchers have investigated various pharmacological strategies in both preclinical and clinical settings. Current clinical interventions prioritize early treatment to reduce the severity of neurological complications. However, plant-derived phytochemicals have the potential to be preventative agents for neurotoxicity. Each herb contains a diverse array of phytochemicals, with only a select few exhibiting demonstrably neuroprotective effects [5,6]. Cubebin, a dibenzyl butyrolactone lignan, has been isolated from various species of plants and families, such as *Aristolochiaceae*, *Myristicaceae*, *Piperaceae*, and *Rutaceae*. It exhibits several therapeutic activities such as trypanocidal, antimycobacterial, analgesic, anti-inflammatory, and vasodilatory [7,8,9,10,11].

This study aimed to evaluate the neuroprotective effects of cubebin in rats with METH-evoked neurotoxicity, followed by cognitive impairments and hippocampal damage, focusing on its ability to inhibit lipid peroxidation and reduce pro-inflammatory markers.

## 2. Methodology

### 2.1. Animals

During the experiment, Wistar rats (weighing 180 ± 20 g and aged 10–12 weeks) were acquired and given appropriate care, including feeding, housing, and enrichment. The rats were housed under a controlled environment with a 12 h light–dark cycle, a temperature of 24 ± 3 °C, and humidity controlled at 50% to 60%. They were fed well and had free access to water. After their acquisition, the rats were acclimated for 7 days. This experiment was carried out with approval from the Institutional Animal Ethics Committee, adhering to ARRIVE guidelines. The research protocol was approved by the institutional research board of Batterjee Medical College, Jeddah, Saudi Arabia, with approval number (RES-2024-0084).

### 2.2. Drugs and Chemicals

**Figure 1:**
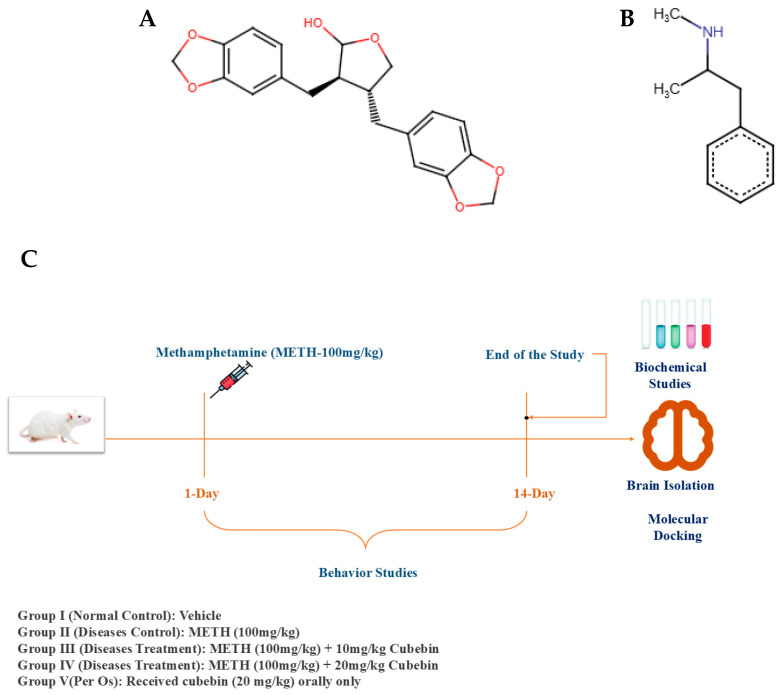
(**A**,**B**) Chemical structure of (**A**) cubebin and (**B**) methamphetamine. (**C**) Experimental design.

METH and cubebin (>98.0% purity) (Figure 1A,B) were soluble in dimethyl sulfoxide (DMSO) and acquired from MSW Pharma, M.S., India. Interleukin-6 (MSW-IL-6), IL-1β (MSW-IL-1β), NF-κB (MSW-NF-κB), BDNF (MSW-BDNF), caspase-3 (MSW-Cap-3), and caspase-9 (MSW-Cap-9) were quantified using commercially available enzyme-linked immunosorbent assay (ELISA) kits (MSW Pharma, Chandrapur, M.S., India). The remaining chemicals used in this experiment were of standard analytical grade.

### 2.3. Acute Toxicity Studies and Prediction of ADMET by Computational Analysis

In accordance with OECD guideline 423, the acute oral toxicity of cubebin was assessed in Wistar rats. Administration of cubebin at doses of 10 and 20 mg/kg by oral gavage did not elicit any signs of toxicity or mortality, thereby indicating a favorable safety margin at these dose levels [12].

### 2.4. Experimental Design

The design of the experimental study is demonstrated in Figure 1C. Thirty rats were randomized and grouped into five clusters (*n* = 6).

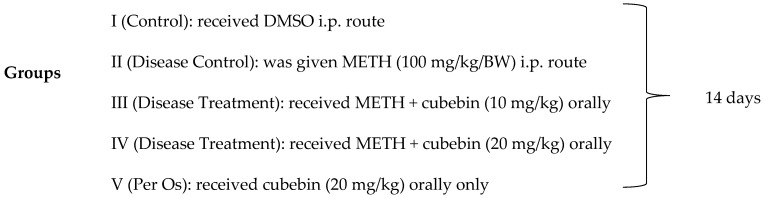



The animals were subjected to behavioral evaluation utilizing the Morris water maze (MWM) and Y-maze tests on the 14th day. Group I (Control) received DMSO via the intraperitoneal (i.p.) route. Group II (Disease Control) was given METH (100 mg/kg/BW) via the i.p. route. Group III (Disease Treatment) received cubebin (10 mg/kg) orally, while group IV (Disease Treatment) received cubebin (20 mg/kg) orally, and group V (Per Os) received cubebin (20 mg/kg) orally only [13]. After behavioral assessment, rats were euthanized by administration of 75 mg/kg of ketamine/10 mg/kg of xylazine. The rats were sacrificed by cervical dislocation for the measurement of biochemical parameters such as neurotransmitters, including norepinephrine (NE), dopamine (DA), gamma-aminobutyric acid (GABA), acetylcholine esterase (AChE), choline-acetyltransferase (ChAT), oxidative stress indicators (malondialdehyde (MDA) and nitric oxide (NO)), antioxidants (catalase (CAT), glutathione (GSH), superoxide dismutase (SOD)), pro-inflammatory markers (IL-6, IL-1β, TNF alpha, NF-κB), BDNF, caspase-3, and caspase-9.

### 2.5. Behavioral Studies

#### MWM Test

Long-term memory and spatial learning were evaluated using the Morris water maze (MWM) test, as described by Aksoz et al. [14]. The apparatus consisted of a circular tank divided into four equal quadrants and filled with opaque water maintained at 24 ± 1 °C using non-toxic creamy milk. A hidden escape platform was submerged 2.0 cm below the water surface and remained in the same quadrant throughout the study. During the acquisition phase, each animal underwent three trials per day, released from different starting points in a randomized manner. Each trial lasted a maximum of 60 s. The time taken to locate the hidden platform (escape latency) was recorded. If an animal failed to find the platform within the allotted time, it was gently guided to the platform and assigned a latency score of 60 s. Following each trial, the animal was allowed to remain on the platform for 20 s. After each session, the animals were towel-dried and returned to their home cages.

On the fifth day, a probe trial was conducted to assess memory consolidation. The platform was removed, and each animal was allowed to swim freely for 60 s. The time spent in the target quadrant, where the platform had been previously located, was recorded as an index of spatial memory retention.

### 2.6. Biochemical Estimations

#### 2.6.1. Brain Tissue Preparation

Separated from the extracted brain tissue, the brain was cleaned with 0.9% (*w*/*v*) normal sterile saline and centrifuged for 15 min at 2000 rpm to obtain a homogenate, which was stored at −80 °C.

For the biochemical estimation, the hippocampus was separated from the extracted brain tissue, cleaned with 0.9% (*w*/*v*) normal sterile saline, and centrifuged for 15 min (2000 rpm) to obtain a homogenate, which was stored at 4 °C for further processing.

#### 2.6.2. Neurotransmitter Levels

The concentrations of neurotransmitters, including NE, DA, and GABA, within brain tissue homogenates were quantified through high-performance liquid chromatography (HPLC) equipped with an Agilent 1100 (Agilent Technologies, Waldbronn, Germany) variable wavelength detector (VWD). ChemStation software (Rev. B.04.03) was utilized for control and subsequently for data analysis.

### 2.7. Antioxidant Enzymes

#### 2.7.1. Superoxide Dismutase (SOD)

A photochemical method utilizing nitroblue tetrazolium (NBT) was employed to assess SOD activity. Each reaction mixture, with a total volume of 1.5 mL, consisted of 100 mM Tris/HCl buffer (pH 7.8), 75 mM NBT, 2 µM riboflavin, and 6 mM EDTA. Following the incorporation of 1.5 mL of the sample supernatant into this mixture, the reaction proceeded through the photochemical generation of superoxide radicals, leading to the reduction of NBT to formazan and subsequent alteration in absorbance. The variation in absorbance change was quantified at 560 nm and represented in units per milligram (U/mg) of protein.

#### 2.7.2. Reduced Glutathione (GSH)

Brain tissue was taken and homogenized in 0.1 M phosphate buffer (pH 7.4) for the determination of GSH levels. The homogenates were then subjected to protein precipitation in a mixture of 20% trichloroacetic acid (TCA) containing 1 mM EDTA solution. Following centrifugation, 200 µL of liquid supernatant was added to newly labeled test tubes. DTNB solution (0.1 mM) known as Ellman’s reagent, was made up of 0.3 M phosphate buffer containing 1% sodium citrate and added at a volume of 1.8 mL. The final volume of each test tube was adjusted to 2 mL with additional buffer. All the samples were then quantified at 412 nm against the blank as previously used in the absorbance experiment. GSH concentration was calculated per milligram of protein (U/mg).

#### 2.7.3. Catalase (CAT)

To estimate CAT activity, the reaction mixture was prepared by combining 100 µL of the supernatant with 150 µL of 0.01 M phosphate buffer at pH 7.0. To start the reaction, 250 µL of 0.16M H_2_O_2_ was added and allowed to react at 37 °C for one minute. The reaction was then terminated by the incorporation of 1.0 mL of a dichromate-acetic acid solution. Following the addition of this reagent, the tubes were subjected to a hot water bath for a duration of 15 min. This incubation period led to the development of a green color, which was subsequently quantified at 570 nm using a spectrophotometer. For accurate determination, a parallel set of tubes without the enzyme was processed under the same conditions. The concentration of catalase, represented in units per milligram (U/mg), was determined by a change in absorbance per unit time.

### 2.8. Oxidative Stress Markers

#### 2.8.1. Malondialdehyde (MDA)

In order to determine the primary indicator of oxidative stress, MDA, we filled each test tube with 500 mL of the diluted homogenate and also prepared a control tube containing 500 mL of Tris-HCl buffer (50 mM, pH 7.4). We added 250 mL of 20% trichloroacetic acid (TCA) and 500 µL of 0.67% thiobarbituric acid (TBA) to each tube. We sealed the tubes using glass beads and placed them in a water bath at 90 °C for 10 min. Once the tubes had cooled to room temperature, they were spun in a centrifuge at a speed of 3000 rpm for 15 min. The sample’s absorbance was determined at 535 nm using the spectrophotometer, and the result was represented in µmol/L.

#### 2.8.2. Nitric Oxide (NO)

The NO production was assessed based on the nitrite concentration, which was quantified utilizing a colorimetric Griess reagent. Griess reagent was made up by incorporating an equal amount of 1% sulfanilamide in 5% phosphoric acid and 0.1% N-(1-naphthyl) ethylenediamine dihydrochloride (NEDD) in water. A 96-well plate was filled with 100 µL of the plasma sample, and subsequently, 100 µL of Griess reagent was incorporated. Subsequently, the plate was incubated at 37 °C for 10 min. Following incubation, the optical density of the microplate was quantified at a wavelength of 540 nm using a spectrophotometer.

### 2.9. Neuroinflammatory Cytokines

The levels of various neuroinflammatory cytokines such as IL-1β, IL-6, TNF-α, NF-kB, cAMP response element-binding protein (CREB), and brain-derived neurotrophic factor (BDNF) were quantified by using commercially available standard ELISA kits. The concentration of these inflammatory cytokines was assessed and expressed in picograms per milliliter (pg/mL) units.

### 2.10. Apoptotic Markers

Caspase-3 and caspase-9, critical mediators of the apoptotic pathway, were quantified using commercially available enzyme-linked immunosorbent assay (ELISA) kits (Merilyzer Eiaquant, Meril Life Sciences Pvt. Ltd., Gujarat, India). These microwell-based assays utilize pre-coated antibodies specific to the target proteins, and the concentrations were expressed in ng/mL.

### 2.11. Molecular Docking (MD)

#### 2.11.1. Target Protein Retrieval and Preparation

X-ray crystallographic structures of the proteins CREB (PDB ID: 4NYX) [15], BDNF (PDB ID: 1B8M) [16], and caspase-3 (PDB ID: 1NME) [17] were acquired from the Protein Data Bank server [18]. These structures were thoroughly assessed by applying several criteria, such as resolution, mutation status, wwPDB validation, co-crystallized ligand, and Ramachandran plots. Table 1 provides a detailed summary of the validation results.

Protein structures were optimized and minimized using UCSF Chimera v1.16. [19]. Force field parameters from AMBER ff14SB were applied to standard residues, while AM1-BCC was used for nonstandard residues. All non-protein components, including water molecules, co-crystallized ligands, and extraneous chains, were removed prior to the calculations.

#### 2.11.2. Grid Generation

AutoDock Tools 1.5.6, Chimera 1.11, and Maestro Version 12.7.161 were utilized to generate and validate the grid [20,21]. Table 2 depicts the active site amino acids, and grid parameters (Table 3) were determined based on the orientation of the co-crystal ligand and the results obtained from the CASTp 3.0 Server. The grid box dimensions were minimized to ensure compatibility with the protein’s active site and the anticipated ligand for docking.

#### 2.11.3. Ligand Preparation

The ligand molecule was sourced from the ChemSpider3 database and imported into the MarvinSketch4 software for structural optimization. The molecule was subjected to 2D and 3D structural cleaning to remove any inconsistencies or errors. Subsequently, the cleaned structure was minimized using the MMFF94 force field to obtain a minimum energy conformation. This optimized structure, in MOL2 format, was selected for subsequent computational analysis.

#### 2.11.4. MD of the Target Protein with Ligands

Ligands and proteins were optimized and subsequently converted to PDBQT format through in-house Bash scripts leveraging AutoDock Tools 1.5.6 and ADFRsuite, respectively. The ligands were fully flexible, while the receptors were held rigid. Docking simulations were conducted using AutoDock Vina 1.2.5, employing a grid spacing of 0.375 Å. The grid box was centered on the target’s active site, allowing for the exploration of potential binding sites. Default configurations were maintained for other parameters. The XYZ coordinates, as detailed in Table 3, were utilized, along with a CPU setting of 23, exhaustiveness of 32, a maximum of 9 binding modes, and an energy range of 3 kcal/mol. Redocking experiments were performed using the same configurations as the initial docking runs.

#### 2.11.5. Visualization

All results retrieved from the AutoDock Vina Processing run were analyzed in Biovia Discovery Studio Visualizer (Discovery Studio Visualizer v21.1.0.20298) and Maestro 12.3 (academic edition) for 2D and 3D interactions.

### 2.12. Molecular Dynamics Simulation (MDS)

MDS was carried out on protein–ligand complexes (1B8M, 1NME, and 4NYX) with cubebin derivatives (Cubebin_1B8M, Cubebin_1NME, and Cubebin_4NYX) using the Desmond 2020.1 simulation package. The OPLS-2005 force field was employed in a 10 Å × 10 Å × 10 Å period boundary solvation box [22,23,24]. The system was solvated with TIP3P water molecules, and subsequently, the system was neutralized with Na^+^ and maintained at a physiological ionic strength of 0.15 M NaCl [25].

The system was initially equilibrated under NVT conditions for 10 ns, followed by NPT equilibration and minimization for 12 ns. Production runs were carried out for 100 ns under NPT conditions [26]. Temperature and pressure were maintained and controlled using the Nose–Hoover chain coupling scheme and the Martyna–Tuckerman–Klein–Klein chain coupling barostat, respectively [27]. All simulations were conducted at a constant pressure of 1 bar and a varying temperature. A time step of 2 femtoseconds was employed. The Martyna–Tuckerman–Klein chain coupling barostat with a characteristic relaxation time of 2 picoseconds was employed to preserve the pressure. The molecular electrostatic interaction was calculated through a long-range method known as Particle Mesh Ewald [28], with an interaction Coulomb of 9 Å radius.

To evaluate the simulation’s stability, several structural parameters such as the root mean square deviation (RMSD), root mean square fluctuation (RMSF), the radius of gyration (Rg), the number of hydrogen bonds, salt bridges, and the solvent-accessible surface area (SASA) were calculated [29,30].

### 2.13. Binding Free Energy (${∆G}_{bind}$) Analysis

The ${∆G}_{bind}$ and the number of ligand–protein complexes were measured and validated by molecular mechanics and the generalized Born surface area method (MM-GBSA). A total of 50 frames of the MM-GSA ${∆G}_{bind}$ calculation was taken with a 1-frame interval from the MDS. The calculations for these were performed using the thermal_mmgbsa.py script. The additivity principle was used in attempts to quantify the charge of total Gbind (kcal/mol) of Prime MM-GBSA. This principle is the sum of the individual energy terms, Coulombic energy, covalent energy, hydrogen bond energy, van der Waals energy, self-contact energy, lipophilic energy, and solvation energy of the protein and ligand.

The following formula was used to determine ΔG_bind_:

$${∆G}_{bind}={\Delta G}_{MM}+{\Delta G}_{Solv}-{\Delta G}_{SA}$$

where

- ΔG_MM_ represents the difference between the total energies of protein and ligand in isolated form and the free energies of ligand–protein complexes.
- ΔG_Solv_ demonstrates the difference in the GSA solvation energies of the ligand–receptor complex and the addition of the total solvation energies of the receptor and the ligand in the unbound state.
- ΔG_SA_ represents the variation between the ligand and protein surface area energies.

### 2.14. Statistical Analysis

Statistical analyses were performed utilizing GraphPad Prism 9 (GraphPad Software Inc., La Jolla, CA, USA) and demonstrated as mean ± S.E.M. The Shapiro–Wilk test was carried out to determine the normality of the data. For the MWM test, a two-way analysis of variance (ANOVA) test showed significance at *p* ≤ 0.05, followed by the Bonferroni post hoc test. One-way ANOVA and then Tukey’s multiple comparisons test were utilized for the remaining data analysis with a significance level of *p* < 0.05.

## 3. Results

### 3.1. Acute Toxicity Assessment

Cubebin demonstrated a favorable safety profile in acute oral toxicity assessments conducted in rats. Throughout the 14-day observation period, no signs of illness or observable clinical symptoms were detected. The selection of 10 and 20 mg/kg doses of cubebin for the current study was guided by the findings of this acute toxicity evaluation. The ADMET properties of cubebin are summarized in Table 4.

### 3.2. MWM Test

**Figure 2:**
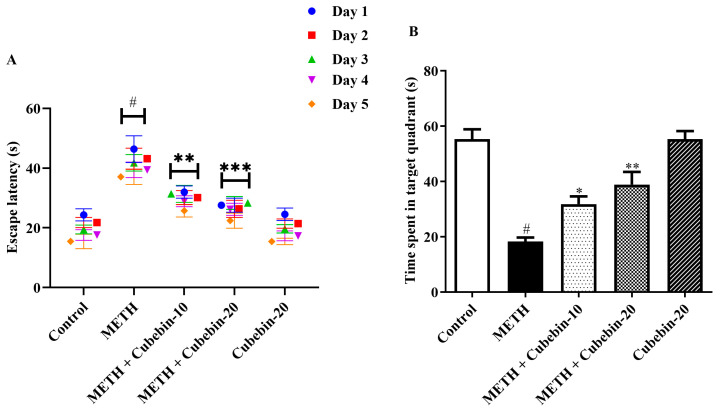
(**A**,**B**) Outcome of cubebin on MWM test. (**A**) Escape latency. (**B**) Time spent in quadrants. Values are expressed as mean ± S.E.M. (*n* = 6). A one-way ANOVA followed by Tukey’s post hoc test; # *p* < 0.001 vs. control; * *p* < 0.05, ** *p* < 0.01, and *** *p* < 0.001 vs. METH.

Figure 2A,B demonstrate the ameliorative effects of cubebin on METH-evoked neurotoxicity in the MWM test. METH-treated rats exhibited significant impairments in spatial learning and memory, as shown by high escape latency and lowered time spent in the target quadrant compared to controls. Notably, cubebin (10 and 20 mg) significantly attenuated escape latency [F (4, 125) = 7.732, *p* < 0.0001] and increased time spent in the target quadrant [F (4, 25) = 24.28, *p* < 0.0001], suggesting a potential neuroprotective role against METH-induced neurotoxicity. Additionally, statistical comparisons between cubebin-treated groups and the control group revealed no significant differences, supporting the conclusion that cubebin restored behavioral performance close to baseline. Notably, cubebin alone (20 mg/kg) did not result in any significant difference compared to the control group, suggesting that cubebin does not adversely affect cognitive performance in healthy rats. Furthermore, rats treated with cubebin alone showed a statistically significant improvement compared to the METH group, reinforcing its protective efficacy (Supplementary Table S1).

### 3.3. Biochemical Estimations

#### Neurotransmitter Levels

**Figure 3:**
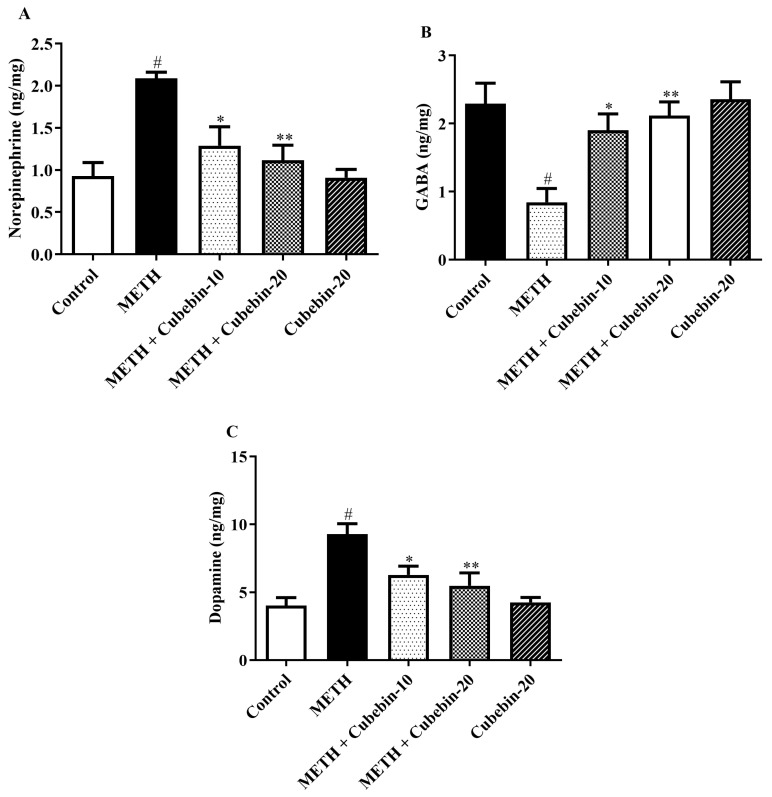
(**A**–**C**) Outcome of cubebin on neurotransmitter levels. (**A**) NE, (**B**) GABA, and (**C**) DA. Values are expressed as mean ± S.E.M. (*n* = 6). A one-way ANOVA followed by Tukey’s post hoc test; # *p* < 0.001 vs. control; * *p* < 0.05 and ** *p* < 0.01 vs. METH.

Figure 3A–C illustrate the outcome of cubebin on neurotransmitters (NE, DA, and GABA) in METH-induced memory dysfunction. Memory dysfunction induced by METH may cause an elevation in levels of NE and DA, while a reduction in GABA is associated with the control group. Conversely, treatment with cubebin at both doses causes a decrease in neurotransmitters such as NE [F (4, 25) = 9.482, *p* < 0.0001] and DA [F (4, 25) = 9.465, *p* < 0.0001] while increasing GABA [F (4, 25) = 6.404, *p* = 0.0011]. These findings may suggest that cubebin has the potential to restore the levels of NE, DA, and GABA (Supplementary Table S2).

### 3.4. Antioxidant Enzymes

**Figure 4:**
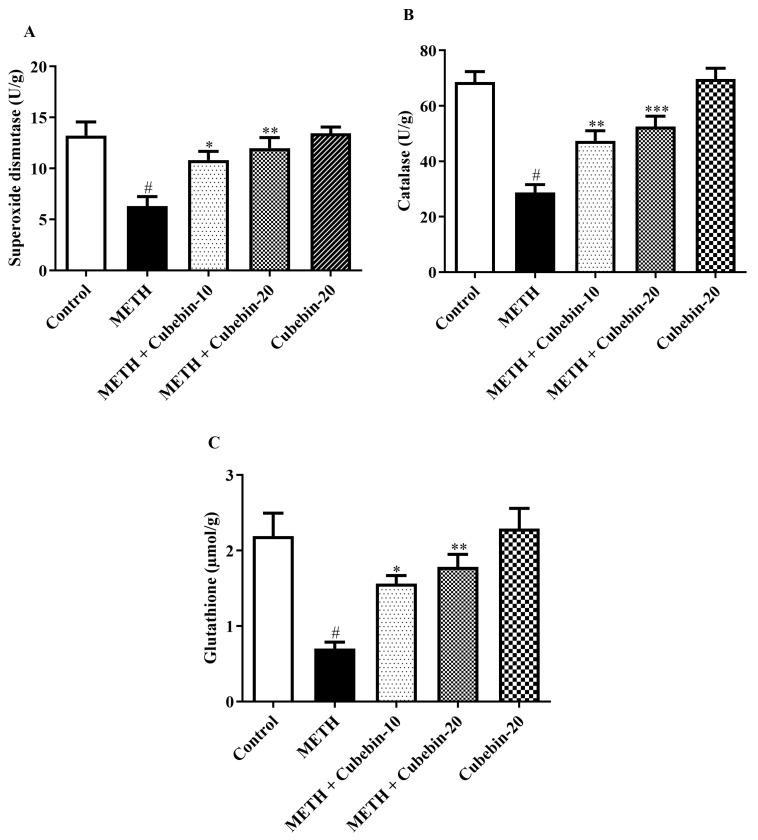
(**A**–**C**) Outcome of cubebin on antioxidant enzymes (**A**) SOD, (**B**) CAT, (**C**) GSH. Values are expressed as mean ± S.E.M. (*n* = 6). A one-way ANOVA followed by Tukey’s post hoc test; # *p* < 0.001 vs. control; * *p* < 0.05, ** *p* < 0.01, and *** *p* < 0.001 vs. METH.

To assess the possible antioxidant effect of cubebin on METH-induced neurotoxicity in rats, the contents of SOD, CAT, and GSH were measured. As shown in Figure 4A–C, the content of SOD, CAT, and GSH was markedly reduced in brain homogenates of METH-injected rats compared with the control group, suggesting METH-evoked oxidative deficits in the brain. These SOD [F (4, 25) = 8.795, *p* = 0.0001], CAT [F (4, 25) = 22.18, *p* < 0.0001], and GSH [F (4, 25) = 9.614, *p* < 0.0001] alterations induced by METH were significantly ameliorated by cubebin treatment at both doses, suggesting that the antioxidative activity of cubebin is responsible for its neuroprotective effect in METH-evoked neurotoxicity. Notably, the cubebin-alone-treated group did not show any significant difference in the levels of NE, DA, and GABA compared to the control group (Supplementary Table S3).

### 3.5. Oxidative Stress Markers

**Figure 5:**
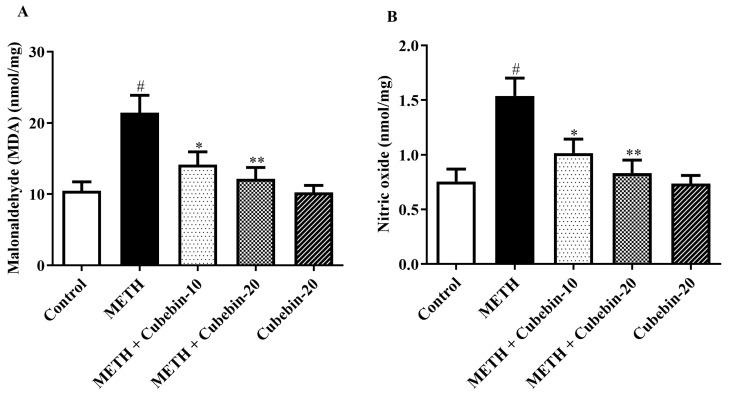
(**A,B**) Outcome of cubebin on oxidative stress markers. (**A**) MDA and (**B**) NO. Values are expressed as mean ± S.E.M. (*n* = 6). A one-way ANOVA followed by Tukey’s post hoc test; # *p* < 0.001 vs. control; * *p* < 0.05 and ** *p* < 0.01 vs. METH.

The effect of cubebin on MDA and NO as indices of oxidative stress is presented in Figure 5A,B. The levels of MDA were increased in the METH group compared to the control group. Cubebin (10 and 20 mg/kg) decreased the MDA [F (4, 25) = 7.415, *p* = 0.0004] and NO [F (4, 25) = 7.425, *p* = 0.0004] levels of the brain. The reduction in MDA and NO as a result of the administration of cubebin indicates that cubebin has a preventive effect against neurotoxicity. The cubebin-alone-treated group did not exhibit any significant difference in MDA and NO levels compared to the control group (Supplementary Table S4).

### 3.6. Neuroinflammatory Cytokines

**Figure 6:**
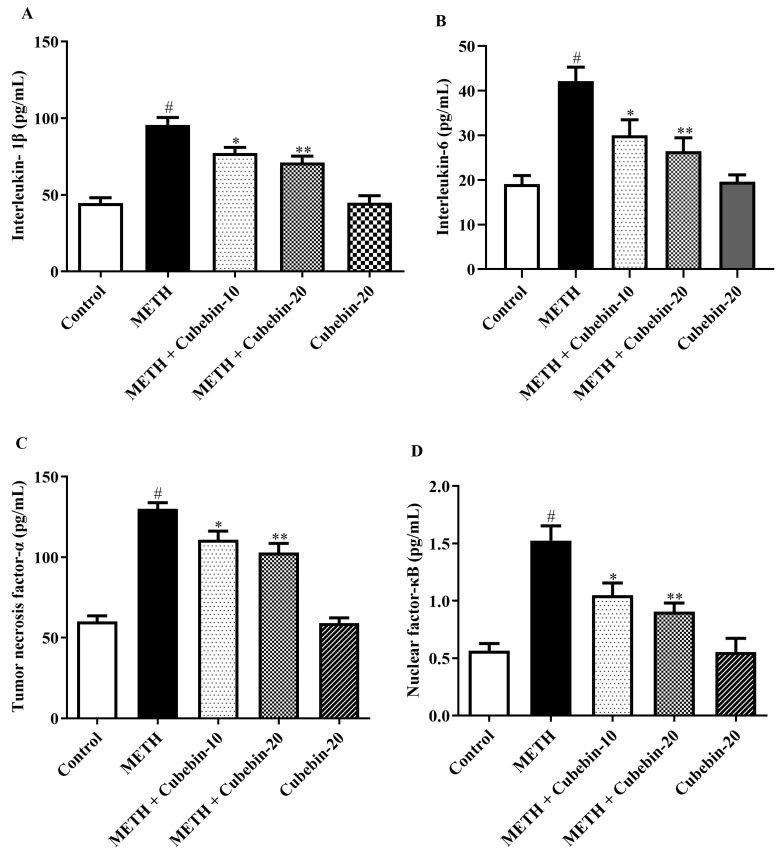

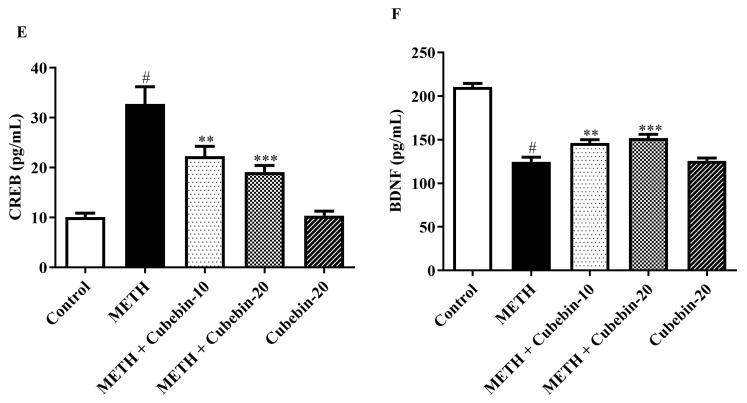
(**A**–**F**): Outcome of cubebin on neuroinflammatory cytokines (**A**) IL-1β, (**B**) IL-6, (**C**) TNF-α, (**D**) NF-kB, (**E**) CREB, and (**F**) BDNF. Values are expressed as mean ± S.E.M. (*n* = 6). A one-way ANOVA followed by Tukey’s post hoc test; # *p* < 0.001 vs. control; * *p* < 0.05, ** *p* < 0.001, and *** *p* < 0.0001 vs. METH.

The effect of cubebin on the indices of different neuroinflammatory cytokines is shown in Figure 6A–F. Exposure of the rats to METH significantly increased the pro-inflammatory cytokines IL-1β, IL-6, TNF-α, CREB, and BDNF in the brain tissue associated with the control group. In contrast, cubebin (10 and 20 mg/kg) markedly decreased the IL-1β [F (4, 25) = 27.13, *p* < 0.0001], IL-6 [F (4, 25) = 12.08, *p* < 0.0001], TNF-α [F (4, 25) = 50.09, *p* < 0.0001], NF-kB [F (4, 25) = 15.53, *p* < 0.0001], CREB [F (4, 25) = 23.34, *p* < 0.0001], and BDNF [F (4, 25) = 66.86, *p* < 0.0001] levels. These results show that the cubebin treatment mitigated neuroinflammatory cytokines in METH-induced neurotoxicity. Moreover, the cubebin-alone-treated group showed no significant difference in the levels of these cytokines compared to the control group (Supplementary Table S5).

### 3.7. Apoptotic Markers

**Figure 7:**
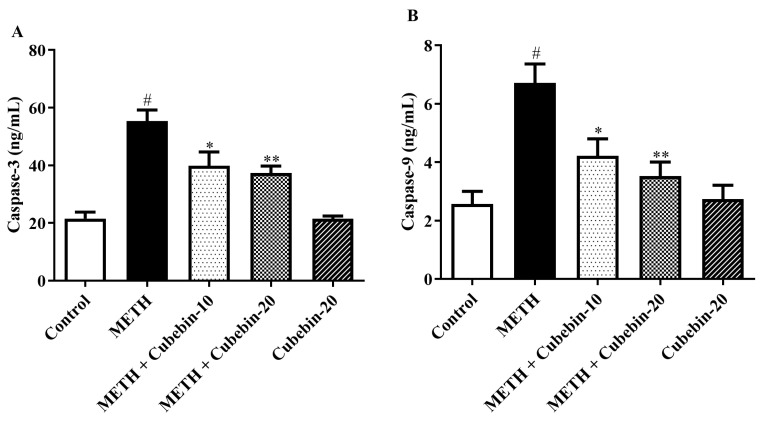
(**A,B**) Outcome of cubebin on apoptotic markers (**A**) caspase-3 and (**B**) caspase-9. Values are expressed as mean ± S.E.M. (*n* = 6). A one-way ANOVA followed by Tukey’s post hoc test; # *p* < 0.001 vs. control; * *p* < 0.05, and ** *p* < 0.001 vs. METH.

The effect of cubebin on the caspase indices, used as apoptotic markers, is shown in Figure 7A,B. According to the caspase-3 and caspase-9 immunohistochemistry staining data, the expression of caspase-3 and caspase-9 is markedly upregulated in the METH group compared to the control. However, cubebin (10 mg and 20 mg) showed a significant downregulation in caspase-3 [F (4, 25) = 20.45, *p* < 0.0001] and caspase-9 [F (4, 25) = 10.17, *p* < 0.0001] in the METH group. Additionally, the cubebin-alone-treated group did not exhibit any significant difference in caspase-3 and caspase-9 expression compared to the control group (Supplementary Table S6).

### 3.8. MD

**Figure 8:**
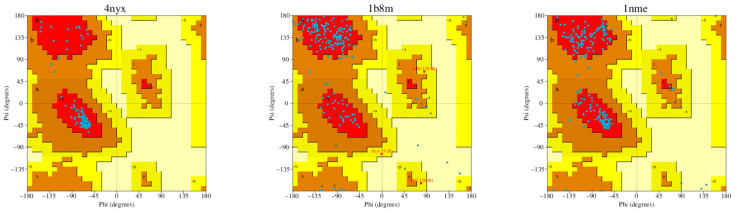
Ramachandran plots for 4NYX, 1B8M, and 1NME obtained from PROCHECK server.

**Figure 9:**
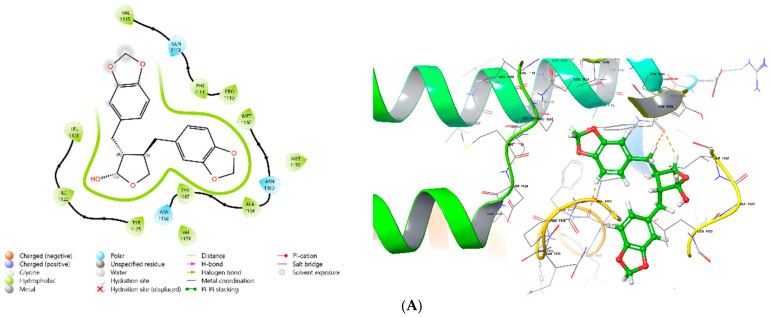

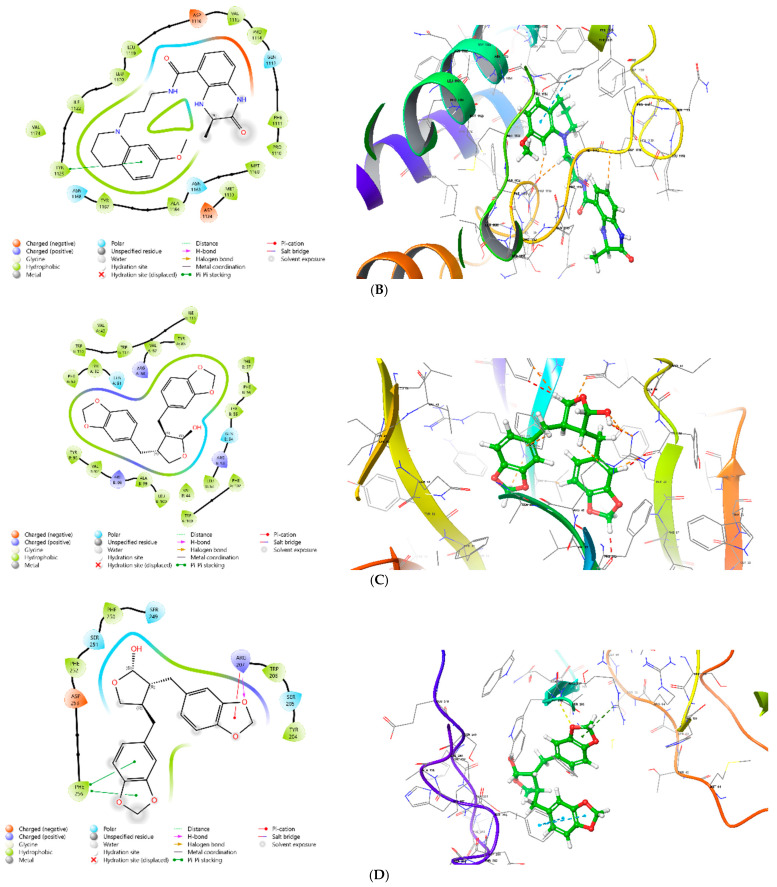

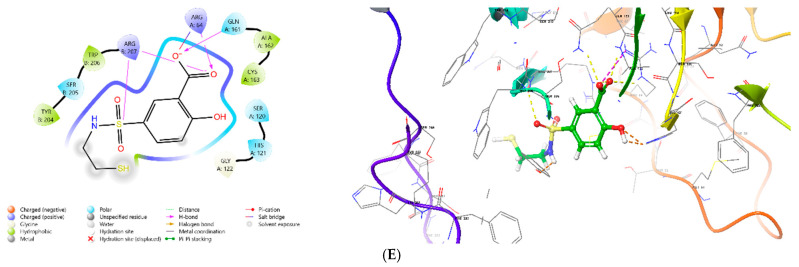
(**A**) The 2D and 3D structure of the interaction of protein 4NYX with cubebin. (**B**) The 2D and 3D structure of the interaction of protein 4NYX with 2O4. (**C**) The 2D and 3D structure of the interaction of protein 1B8M with cubebin. (**D**) The 2D and 3D structure of the interaction of protein 1NME with cubebin. (**E**) The 2D and 3D structure of the interaction of protein 1NME with 159.

Cubebin exhibited strong binding affinities with the selected targets, particularly CREB, BDNF, and caspase-3, with the highest affinity observed for BDNF (−9.173), followed by CREB (−8.881) and caspase-3 (−7.322). Ramachandran plots for 4NYX, 1B8M, and 1NME, generated using the PROCHECK server, are presented in Figure 8. Additionally, 2D and 3D visualizations of the interactions between these proteins and cubebin are demonstrated in Figure 9A–E, with a specific focus on the interaction between 4NYX and cubebin. Table 5 and Table 6 presents the docking scores and intermolecular interactions of ligands targeting CREB (protein ID 4NYX), BDNF (protein ID 1B8M), and caspase-3 (protein ID 1NME), as determined by molecular docking simulations and PLIP analysis, respectively.

### 3.9. MDS

**Figure 10:**
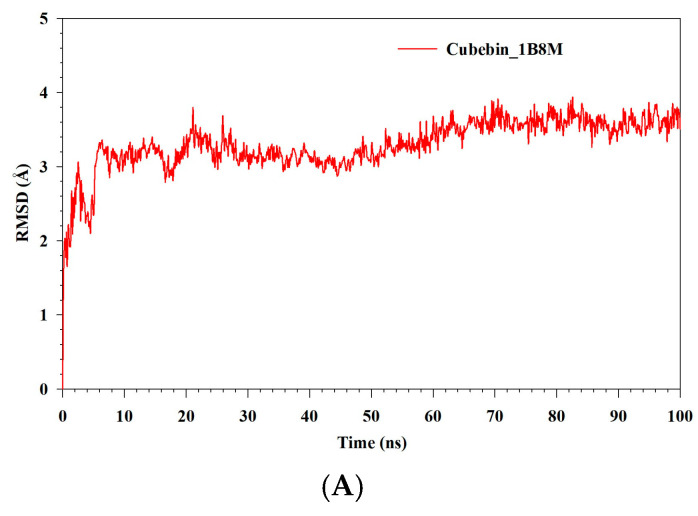

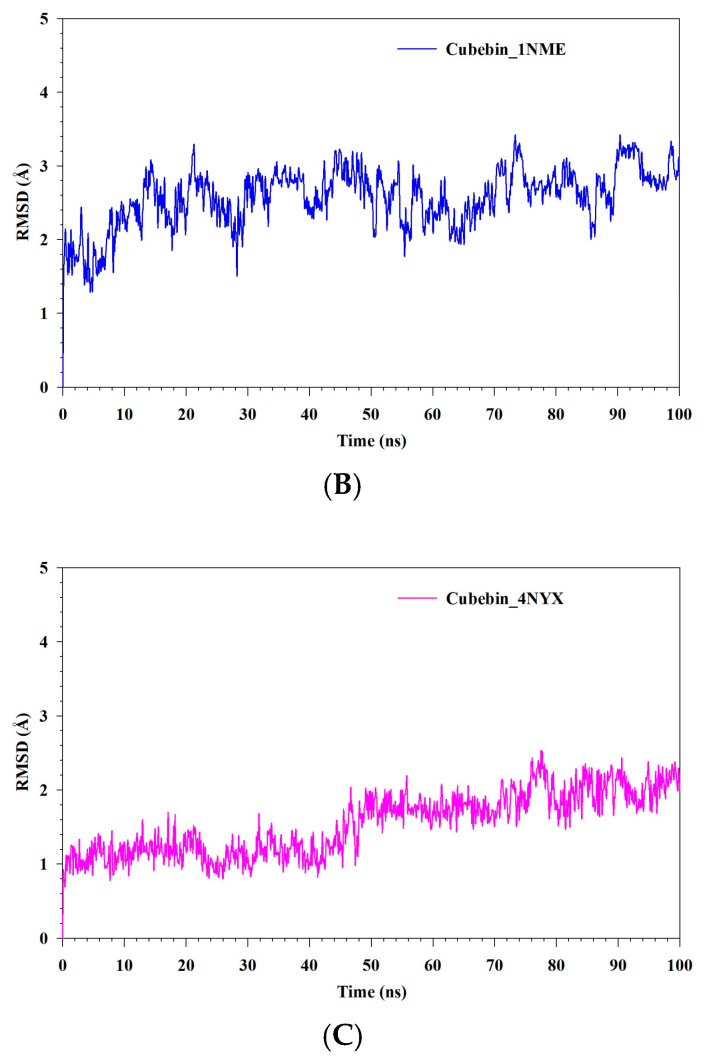
(**A**–**C**) MDS analysis of 100 ns trajectories of RMSD of Cα backbone of (**A**) Cubebin_1B8M, (**B**) Cubebin_1NME, (**C**) Cubebin_4NYX.

**Figure 11:**
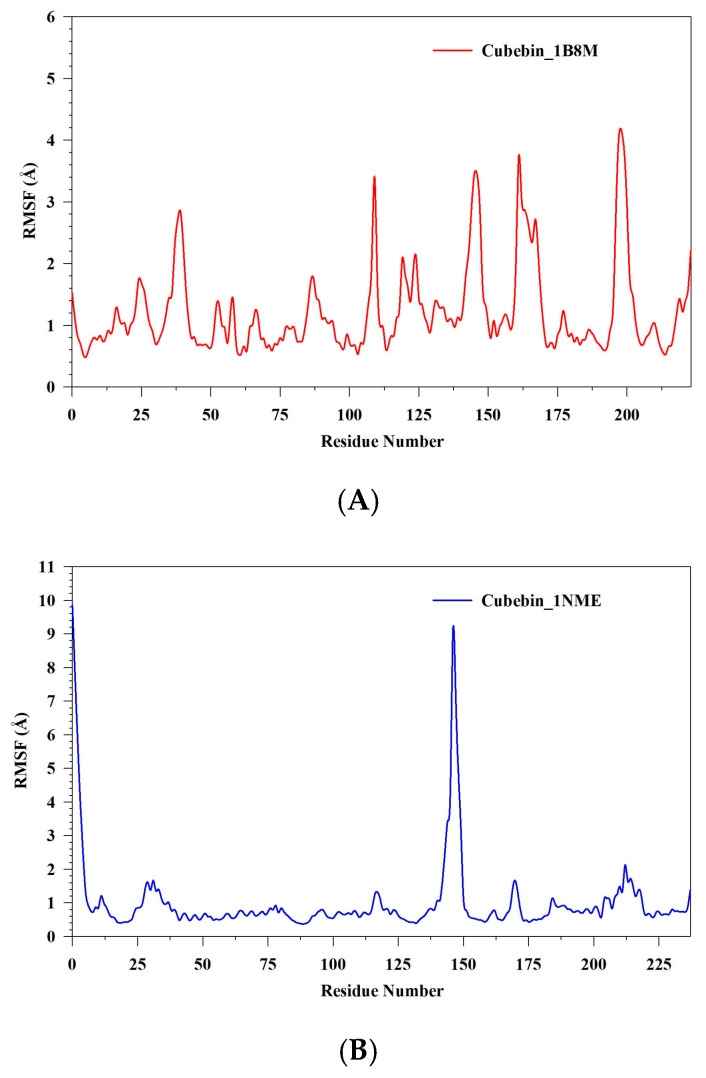

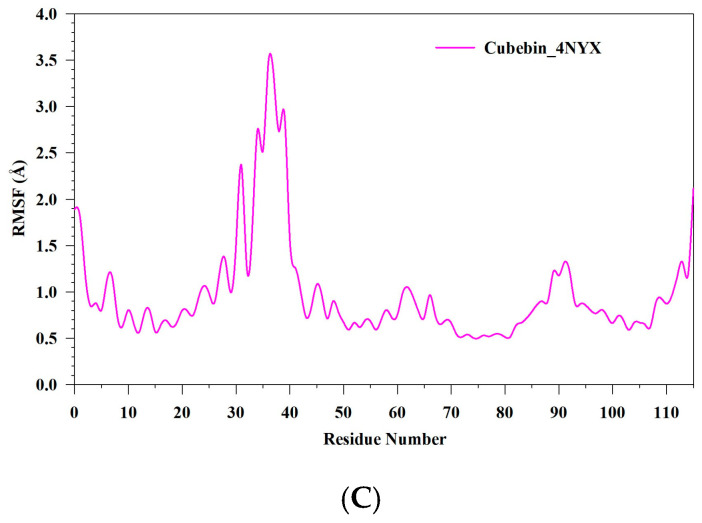
(**A**–**C**) MDS analysis of 100 ns trajectories of RMSF of Cα backbone of (**A**) Cubebin_1B8M, (**B**) Cubebin_1NME, (**C**) Cubebin_4NYX.

MDS was utilized to investigate the stability and convergence of 1B8M, 1NME, and 4NYX in complex with cubebin (denoted as Cubebin_1B8M, Cubebin_1NME, and Cubebin_4NYX, respectively). RMSD analysis, depicted in Figure 10A–C, provides the dynamic behavior of these complexes. Initially, Cubebin_1B8M exhibits an RMSD increase of approximately 3 Å within the first 5 ns, reflecting an adjustment to the simulation environment. The RMSD analysis provides the dynamic behavior and stability of the cubebin–protein complexes. The Cubebin_1B8M complex exhibits an initial rapid RMSD increase followed by stabilization around 3.30 Å, suggesting a relatively stable complex with minimum fluctuations. The Cubebin_1NME complex demonstrates a more dynamic behavior, with an initial increase to 3.42 Å, followed by fluctuations around an average of 2.57 Å. This indicates inherent flexibility within the complex without significant destabilization. The Cubebin_4NYX complex showed a distinct RMSD profile. An initial increase to 1.5 Å within 10 ns corresponds to an equilibration phase. Subsequently, the RMSD stabilizes around 1.54 Å for 40 ns, indicating a well-defined and stable complex. Beyond 45 ns, a gradual increase in RMSD with fluctuations suggests a slight degree of flexibility while maintaining overall stability. Based on the RMSD analysis, the Cubebin_4NYX complex demonstrates the highest degree of stability and structural integrity compared to Cubebin_1B8M and Cubebin_1NME. This is evident from its lower and more consistent RMSD values throughout the simulation. RMSF analysis was conducted on three protein–ligand complexes: Cubebin_1B8M, Cubebin_1NME, and Cubebin_4NYX. The results are visualized in Figure 11A–C. The Cubebin_1B8M complex displayed significant flexibility, with an average RMSF of 1.22 Å. Residues 109, 145–146, 161–162, and 197–199 exhibited pronounced fluctuations, suggesting regions of conformational variability. In contrast, the Cubebin_1NME complex demonstrated overall rigidity, with an average RMSF of 1 Å. However, specific regions, such as residues 0–3 and 144–149, exhibited substantial flexibility, with RMSF values reaching up to 9.85 Å.

The Cubebin_4NYX complex exhibited an average RMSF of 0.98 Å, indicating a relatively rigid structure. Nevertheless, distinct peaks in the RMSF profile, particularly around residues 36–37, with a maximum of 3.47 Å, suggest localized flexibility. Overall, the Cubebin_4NYX complex exhibited the highest degree of structural stability compared to the other two complexes.

**Figure 12:**
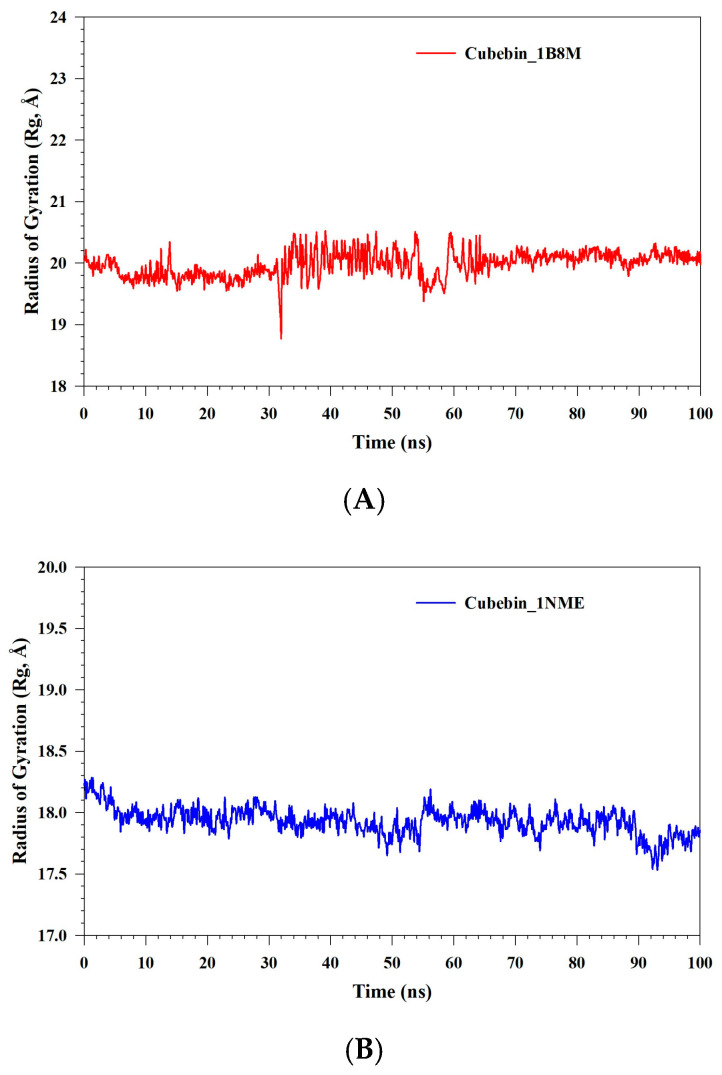

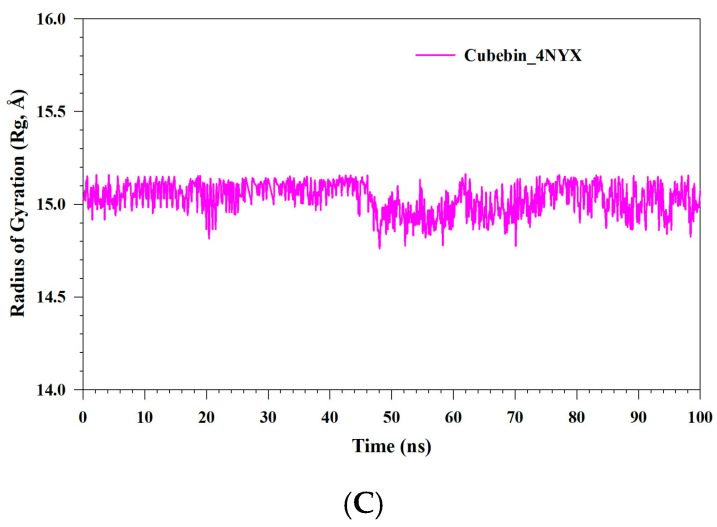
(**A**–**C**) MDS analysis of 100 ns trajectories of Rg of Cα backbone of (**A**) Cubebin_1B8M, (**B**) Cubebin_1NME, (**C**) Cubebin_4NYX.

The radius of gyration (Rg) analysis for the Cubebin_1B8M, Cubebin_1NME, and Cubebin_4NYX complexes is depicted in Figure 12A–C. The Cubebin_1B8M complex exhibited Rg values ranging from 18.84 Å to 20.50 Å, indicating a compact structure with subtle fluctuations over the simulation timeframe. A marked increase in structural dynamism was found between 30 and 60 ns, characterized by substantial fluctuations in the radius of gyration (Rg). Conversely, the Cubebin_1NME complex exhibited a rigid and stable conformation, with Rg values remaining within a narrow range of 17.54–18.28 Å. The Cubebin_4NYX complex maintained a consistently compact structure throughout the simulation, with Rg values fluctuating minimally between 14.78 and 15.15 Å. This limited variation indicates a stable and well-defined complex conformation over the simulated timeframe.

**Figure 13:**
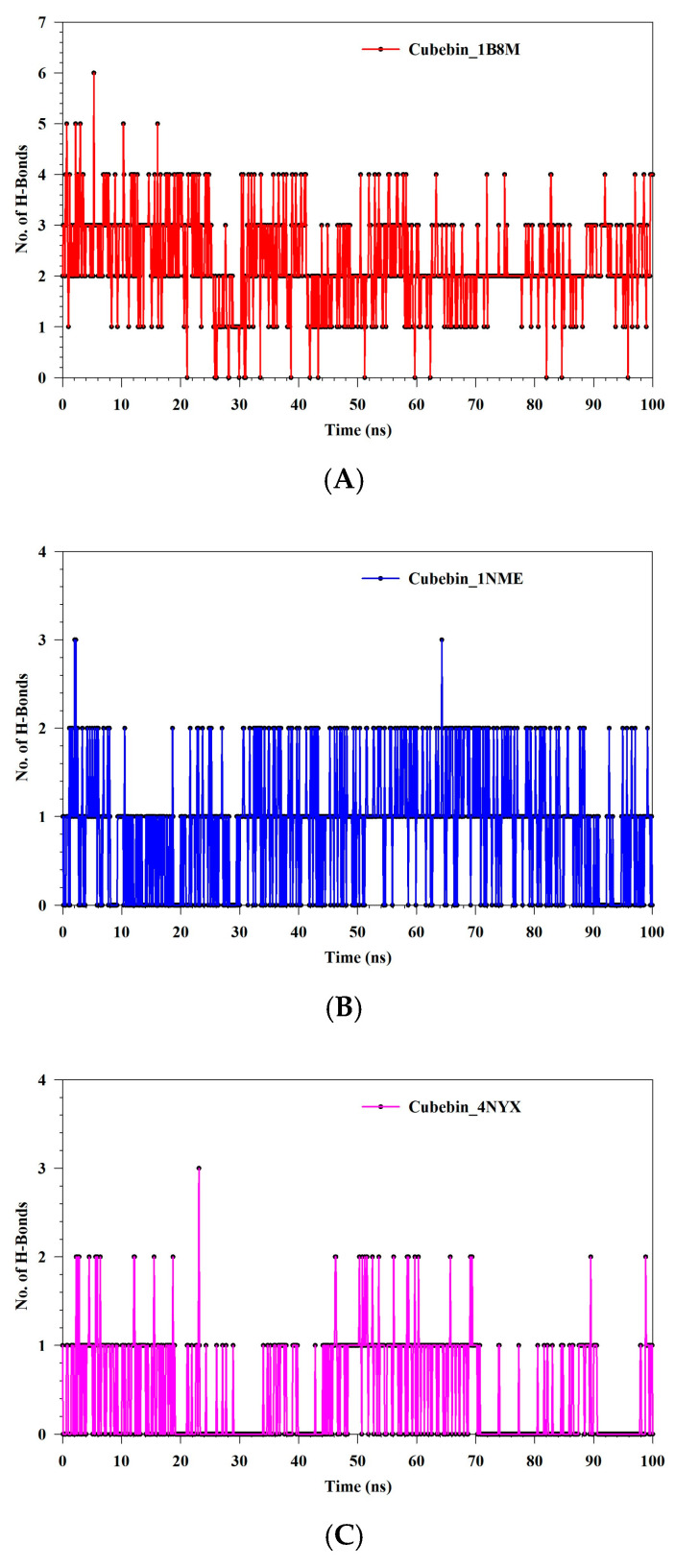
(**A**–**C**) MDS analysis: formation of hydrogen bonds in (**A**) Cubebin_1B8M, (**B**) Cubebin_1NME, (**C**) Cubebin_4NYX.

Figure 13A–C present a comparative analysis of hydrogen bond (H-bond) formation dynamics across various complexes, offering insights into their structural stability. The Cubebin_1B8M complex exhibits a broader range of H-bond fluctuations, varying from 0 to 6 H-bonds with an average of 2.26, suggesting a relatively stable and robust complex. Conversely, the Cubebin_1NME complex displays a narrower range of H-bond fluctuations, varying from 0 to 3 H-bonds with an average of 0.88, indicating weaker or more transient interactions. This suggests a less stable or more dynamic nature of the interactions within this complex. The Cubebin_4NYX complex demonstrates the lowest average H-bond count, fluctuating between 0 and 3 with an average of 0.44, further supporting the notion of weak interactions within this complex.

**Figure 14:**
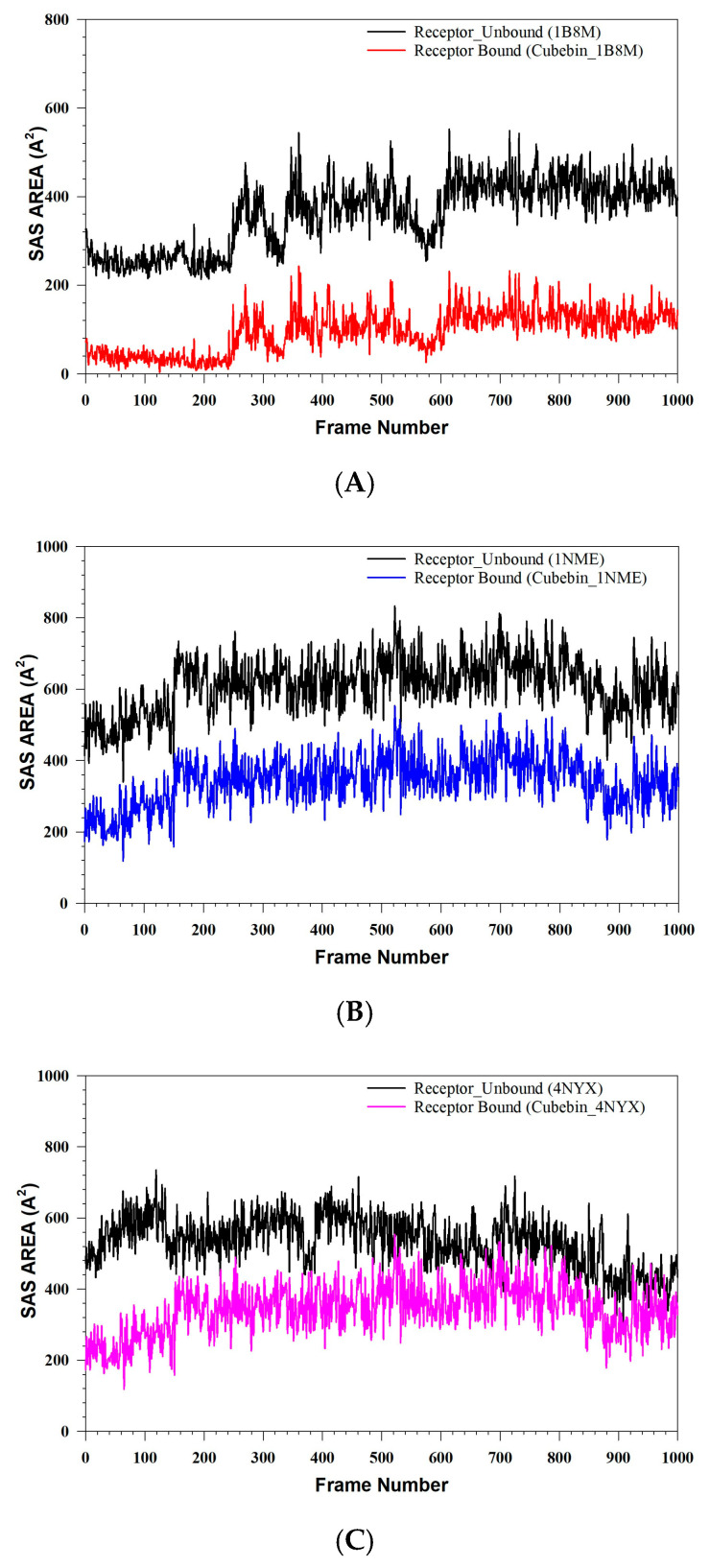
(**A**–**C**) MDS analysis of 1000 Framework of (**A**) SASA of Cubebin_1B8M, (**B**) SASA of Cubebin_1NME, (**C**) SASA of Cubebin_4NYX.

Figure 14A–C present a SASA analysis, illustrating conformational alterations and stability of cubebin upon binding to three distinct proteins: Cubebin_1B8M, Cubebin_1NME, and Cubebin_4NYX. In the Cubebin_1B8M complex, the reduced SASA of the bound receptor relative to its unbound state signifies that cubebin binding effectively diminishes the receptor’s solvent exposure. This decrease in SASA upon binding further implies a stable interaction between the receptor and cubebin.

**Figure 15:**
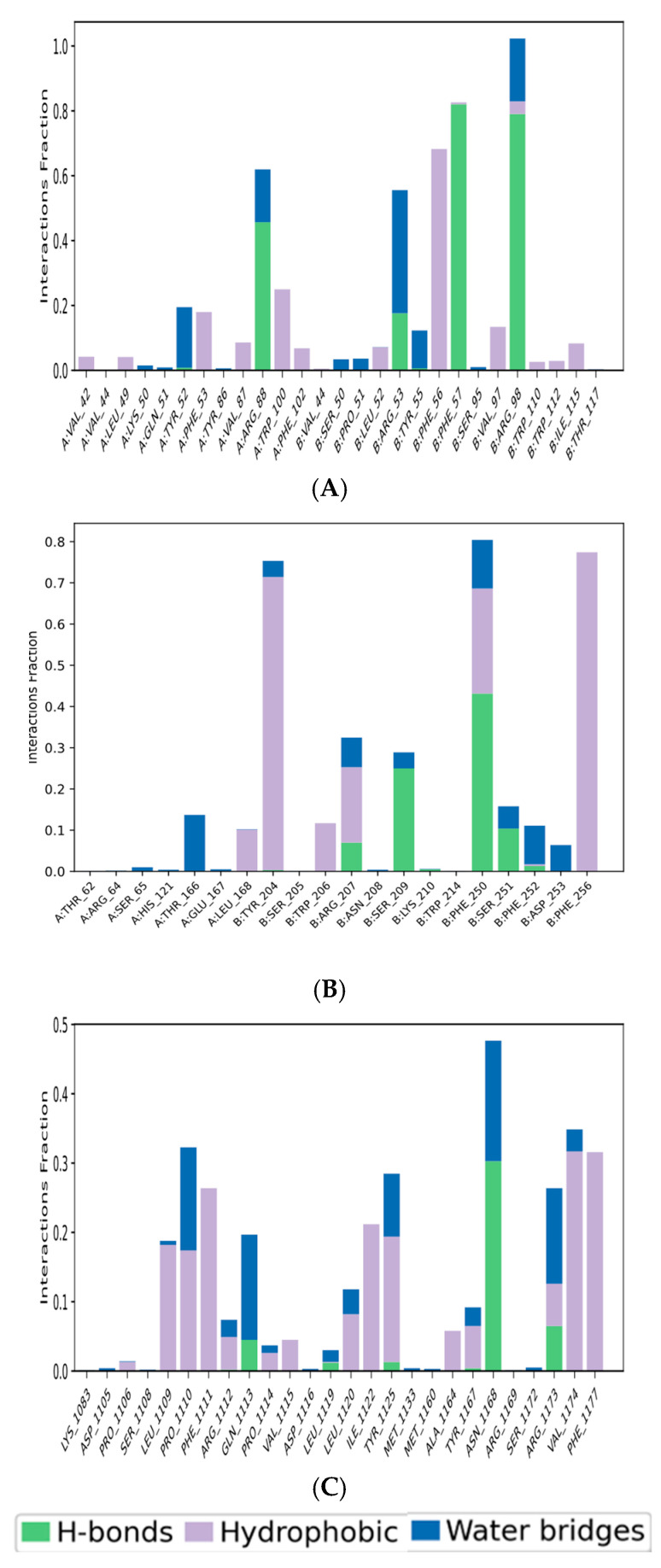
(**A**–**C**) Bar graph of protein–ligand contacts of (**A**) Cubebin_1B8M, (**B**) Cubebin_1NME, and (**C**) Cubebin_4NYX showing the interaction fraction of amino acid residues over the period of simulation.

Protein–ligand interactions within the complexes were investigated through 100 ns molecular dynamics simulations. Interaction types, including hydrogen bonds, hydrophobic interactions, ionic interactions, and water bridges, were categorized and normalized over the simulation trajectories. For each interaction type, the series of contacts between the protein and ligand was quantified, with values ≥ 1 indicating multiple contacts. Figure 15A–C depict bar graphs illustrating the distribution of interaction types across the protein residues over the 100-nanosecond simulation period. The results indicate that hydrogen bonding, ionic interactions, and water bridges were the predominant interaction types observed for the Cubebin_1B8M, Cubebin_1NME, and Cubebin_4NYX protein–ligand complexes.

**Figure 16:**
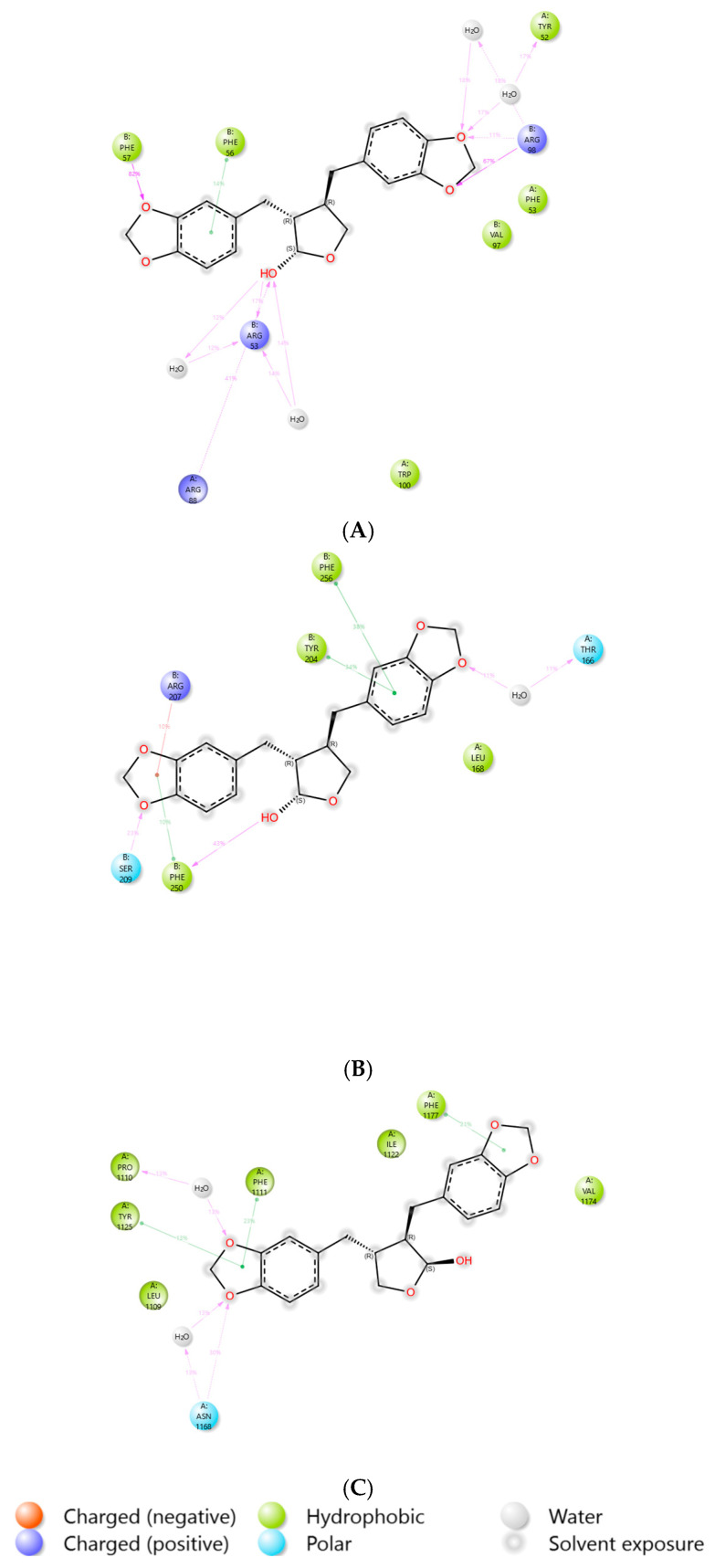
(**A**–**C**) Protein–ligand percent interactions of (**A**) Cubebin_1B8M, (**B**) Cubebin_1NME, (**C**) Cubebin_4NYX.

Figure 16A quantifies the contributions of various interactions to the stability of the Cubebin_1B8M complex. Hydrogen bonding plays a significant role, with residues PHE57 (82%), ARG53 (17%), ARG88 (41%), and ARG98 (78%) participating. Water-mediated interactions, involving ARG53 (52%), TYR52 (34%), and ARG98 (36%), further stabilize the complex. Hydrophobic interactions, primarily driven by PHE56 (14%), also demonstrated optimum binding affinity. Figure 16B illustrates the distribution of protein–ligand interactions within the Cubebin_1NME complex. The complex forms water bridges with THR166 (22%), hydrogen bonds with PHE250 (43%) and SER209 (23%), ionic interactions with ARG207 (10%), and hydrophobic interactions with PHE250 (10%), TYR204 (34%), and PHE256 (38%).

Figure 16C illustrates the distribution of protein–ligand interactions within the Cubebin_4NYX complex. ASN1168 and PRO1110 residues contribute 26% each, forming water bridges with the ligand. Hydrophobic interactions are facilitated by TYR1125 (13%), PHE1111 (23%), and PHE1177 (21%).

### 3.10. Calculations of MM-GBSA

Leveraging MDS, we determined the ΔGbind and constituent energy components for the cubebin–protein complexes (Cubebin_1B8M, Cubebin_1NME, Cubebin_4NYX) using the MM-GBSA approach. Table 7 reveals that Cubebin_1B8M (−75.50 ± 5.29 Kcal/mol) exhibits the strongest binding affinity, followed by Cubebin_1NME (−43.76 ± 5.10 Kcal/mol) and Cubebin_4NYX (−38.81 ± 4.71 Kcal/mol). The enhanced binding of Cubebin_1B8M is attributed to significant contributions from Coulombic, lipophilic, and van der Waals interactions. Cubebin_1NME displays moderate binding affinity with weaker Coulombic and lipophilic interactions, while Cubebin_4NYX exhibits the weakest affinity due to less favorable contributions from all energy components. Overall, these results indicate that electrostatic and van der Waals forces are the main contributors to binding energy in all complexes, while lipophilic interactions and covalent bonds contribute less or more variably. Packing interactions are slightly favorable; however, the solvation energy points to a desolvation cost during the binding process.

## 4. Discussion

This study aimed to assess the neuroprotective effect of cubebin against METH-evoked neurotoxicity in rats. METH, which belongs to the psychostimulant group, is widely recognized as having neurotoxic effects. Even though the detailed pathways by which METH causes neurotoxic effects have not been debated, it is widely postulated that oxidative stress, inflammation, and apoptosis have significant roles to play [31,32]. METH exposure has also been associated with abnormal learning and memory defects that are manifested by poor performance in behavioral tests such as the MWM. These behavioral alterations are typically associated with biochemical changes, including disturbance of neurotransmitter systems and heightened oxidative stress, including inflammation in the brain. An understanding of the pathways through which METH affects the nervous system can provide the foundation on which to base new interventions for the prevention and treatment of the neurological damage associated with METH use. Several human and animal investigations have established that METH neurotoxicity results in cognitive dysfunction. To assess these cognitive impairments, the MWM task, which has been widely used in rodents as a behavioral model, is used. Due to the greater emphasis on learning and memory in the MWM, it is effective for studying age-related neurodecline, stroke-elicited neurological deficits, and neurodegenerative diseases and testing the effectiveness of potential drug treatments [33,34]. In this study, rats demonstrated a significant delay in escape latency during MWM trials. This impairment was further exacerbated by METH administration, as evidenced by prolonged escape latencies and reduced exploration of the target quadrant. Treatment with cubebin-10 and -20 significantly reduced escape latency by 66.3% and 45.0% (from 37.06 ± 2.51 to 25.71 ± 2.09; 22.42 ± 2.52 s; *p* < 0.0001) and improved time spent in the target quadrant by 42.6% and 29.7% (from 18.24 ± 1.48 to 31.77 ± 2.82; 38.88 ± 4.57 s; *p* < 0.0001) compared to the METH group. These quantitative improvements are in line with a previous finding [35].

Neurotoxicity also arises from alterations in neurotransmitter systems, including abnormal release, modified concentration or residence time, or interference with postsynaptic receptor function as an agonist or antagonist. Neurotransmitters are endogenous signaling molecules that modulate and propagate cellular communication, critically impacting brain function, behavior, and cognition [2]. METH primarily increases extracellular monoamine levels, including DA and NE, by inhibiting their reuptake and stimulating their release. METH’s structural similarity to catecholamines facilitates its interaction with their transporters, leading to elevated intracellular levels. Notably, METH induces significant NE release, which may directly contribute to its behavioral and neurotoxic effects. NE modulates DA activity, influencing reward pathways and potentially underlying METH addiction. Additionally, genetic variations affecting NE synthesis may contribute to individual susceptibility to METH-induced behaviors [36,37].

GABA acts as the primary inhibitory neurotransmitter within the central nervous system. Its synthesis from glutamate is catalyzed by a specialized enzyme exclusively expressed in GABAergic neurons. GABAergic signaling serves a crucial role in the regulation and synchronization of neuronal activity within the hippocampus, a brain region crucial for memory formation and function. This region is particularly vulnerable to early structural and functional impairments associated with neurological disorders [38,39]. METH administration in rats induced neurotoxicity followed by memory impairment and dysregulation of neurotransmitter levels, characterized by elevated levels of NE and DA and decreased levels of GABA compared to controls. Treatment with cubebin at both doses significantly restored neurotransmitter balance by reducing NE levels by 38.3% and 46.6% (from 2.088 ± 0.07 to 1.288 ± 0.22; 1.115 ± 0.17 ng/mg; *p* < 0.001), and DA levels by 32.3% and 40.9% (from 9.280 ± 0.76 to 6.285 ± 0.64; 5.482 ± 0.94 ng/mg; *p* < 0.001), while increasing GABA levels by 125.5% and 151.0% (from 0.8433 ± 0.20 to 1.902 ± 0.23; 2.117 ± 0.20 ng/mg; *p* < 0.001), respectively, compared to the METH group. These neurochemical changes are consistent with a previous study reporting cubebin’s regulatory effect on neurotransmitter balance under neurotoxic conditions [40].

The failure to maintain a balanced antioxidant/oxidant ratio implies that the concentration of ROS is higher than the capacity of the antioxidant systems to scavenge them in the brain. ROS, a class of molecules including free radicals and non-radical oxygen derivatives, are naturally generated by metabolic processes. Excessive ROS production or lowered antioxidant defenses disrupt the redox balance within cells, leading to oxidative stress. The brain’s unique characteristics, such as more consumption of oxygen, plenty of polyunsaturated fatty acids, and limited antioxidant reserves, make it particularly susceptible to oxidative stress. Increased ROS production is directly linked to cellular damage observed in aging and various neurological diseases [32,41,42]. Antioxidant enzymes, including SOD and GSH, play a crucial role in protecting against oxidative damage. They serve as a vital defense mechanism for aerobic organisms, allowing their survival in an oxygen-rich environment. SOD belongs to the group of endogenous enzymes, and it is involved in the dismutation of superoxide anions into oxygen and hydrogen peroxide. Also, GSH helps to prevent the oxidative damage of cells that is brought about by free radicals and other peroxides [31]. Catalase is also an essential antioxidant enzyme that catalyzes hydrogen peroxide into harmless water and oxygen. This enzymatic activity plays a vital role in the suppression of oxidative-stress-related diseases [43]. METH administration in rats significantly decreased antioxidant markers, reducing SOD by 52.3% (from 13.22 ± 1.33 to 6.312 ± 0.93 U/g), CAT by 58.1% (from 68.57 ± 3.78 to 28.73 ± 2.86 U/g), and GSH by 68.0% (from 2.190 ± 0.30 to 0.7000 ± 0.08 U/g; *p* < 0.001), indicating elevated oxidative stress. Treatment with cubebin-10 and -20 significantly restored these enzyme levels, increasing SOD by 71.4% and 90.1% (10.82 ± 0.84; 12.00 ± 1.03 U/g; *p* < 0.001), CAT by 64.9% and 82.8% (47.38 ± 3.61; 52.53 ± 3.73 U/g; *p* < 0.001), and GSH by 123.3% and 154.6% (1.563 ± 0.10; 1.782 ± 0.16 U/g; *p* < 0.001), respectively, compared to the METH group. These antioxidant-restorative effects align with previous evidence supporting cubebin’s role in mitigating oxidative damage [13,44].

The restoration of antioxidant capacity is essential for preserving neuronal health and function. Oxidative stress, indicated by elevated levels of MDA, is a key contributor to neuronal damage and dysfunction [45]. Treatment with cubebin at both doses significantly attenuated lipid peroxidation by reducing MDA levels by 34.1% and 43.3% (from 21.44 ± 2.44 to 14.13 ± 1.80; 12.16 ± 1.58 nmol/mg; *p* < 0.001), respectively, compared to the METH group, suggesting its ability to mitigate oxidative damage caused by METH. These findings reinforce the antioxidant property of cubebin, consistent with a previous report [46]. METH induces the activation of nitric oxide synthase and increases the production of NO in the rodent hippocampus [36]. Treatment with both doses of cubebin significantly lowered NO levels by 34.02% and 45.83%, from 1.538 ± 0.16 to 1.015 ± 0.12 and 0.8333 ± 0.11 nmol/mg, respectively (*p* < 0.0001), compared to the METH group. These study results align with the findings of an earlier report [47].

Neuroinflammation is triggered by METH exposure through the upregulation of pro-inflammatory cytokine production. Astrocytes are the primary brain cells that serve an essential role in regulating both pro- and anti-inflammatory cytokines. Elevated levels of inflammatory markers from astrocytes are associated with various CNS disorders. Reactive astrogliosis, characterized by morphological and functional changes, is a hallmark of neurological conditions like stroke, trauma, and neurodegenerative diseases. Reactive astrocytes contribute significantly to inflammation by producing pro-inflammatory cytokines and chemokines [31,48]. The neuroinflammatory effect is mediated by the activation of microglia through Toll-like receptor 4 (TLR4), which recognizes both endogenous and exogenous ligands. TLR4 activation initiates two signaling pathways: the MyD88-dependent and MyD88-independent pathways. The MyD88-dependent pathway, involving MyD88, TRAF6, and IRAK, leads to activation of NF-κB and subsequent generation of pro-inflammatory cytokines [48]. However, treatment with cubebin significantly attenuated pro-inflammatory cytokines by reducing IL-1β levels by 19.2% and 25.6% (from 95.64 ± 4.84 to 77.26 ± 3.76; 71.13 ± 4.14 pg/mL; *p* < 0.001), IL-6 levels by 28.8% and 37.2% (from 42.17 ± 3.11 to 30.01 ± 3.49; 26.48 ± 2.98 pg/mL; *p* < 0.001), TNF-α levels by 14.8% and 20.9% (from 130.1 ± 3.86 to 110.8 ± 5.49; 102.9 ± 5.70 pg/mL; *p* < 0.001), and NF-κB levels by 31.3% and 40.7% (from 1.525 ± 0.12 to 1.048 ± 0.10; 0.9050 ± 0.07 pg/mL; *p* < 0.001), respectively, compared to the METH group. These findings reinforce the antioxidant property of cubebin, consistent with previous reports [13,49].

Several hippocampal proteins, including BDNF and CREB, play important roles in both neurogenesis and neurodegeneration. This study investigates the interplay of these proteins in these processes. BDNF is a neurotrophic factor responsible for promoting neuronal growth, differentiation, and maturation. It is closely linked to learning and memory processes in rodents, particularly through its regulation of synaptogenesis and synaptic plasticity in the adult CNS [50,51]. CREB, a transcription factor, binds to cAMP response elements and regulates the expression of genes involved in neurogenesis. It can stimulate or suppress the expression of various genes, including BDNF. CREB is a key regulator of genes associated with synaptic and neural plasticity. BDNF and CREB are interconnected. BDNF is a target gene of CREB, and CREB’s transcriptional activity is influenced by BDNF signaling. Both of the proteins are found in different areas of the brain, which are involved in cognitive, emotional, and reward functions. A disruption in the CREB transcriptional pathway has been associated with oxidative stress, apoptotic processes, and neuronal death [51,52]. Treatment with cubebin significantly increased BDNF levels by 17.4% and 21.8%, raising them from 124.6 ± 5.21 pg/mL to 146.3 ± 3.72 pg/mL and 151.8 ± 4.27 pg/mL, respectively (*p* < 0.001). Additionally, cubebin treatment reduced CREB levels by 32.1% and 41.9%, decreasing them from 32.76 ± 3.41 pg/mL to 22.25 ± 1.99 pg/mL and 19.03 ± 1.38 pg/mL, respectively (*p* < 0.001). These findings suggest that cubebin has the potential to normalize the cortical neuroplasticity markers that are dysregulated by METH. Based on this study, we can conclude that cubebin treatment may effectively reverse the changes in BDNF and CREB expression caused by METH.

Apoptosis, known as programmed cell death, occurs due to the activation of many signal transduction pathways that post-translationally modify the cellular macromolecules such as lipids, proteins, and nucleic acids. A characteristic feature of apoptosis is the alteration of the cell membrane and, consequently, the permeability of the cell membrane. Studies demonstrated that cell death by apoptosis may play a role in drug-mediated neurotoxicity, including neurotoxicity mediated by METH and other psychotropic agents [53]. In the present study, METH-induced neurotoxicity significantly increased the levels of caspase-3, a key protease involved in apoptotic cell death. Treatment with cubebin at doses of 10 mg/kg and 20 mg/kg resulted in a significant reduction in caspase-3 levels by 27.8% and 32.6%, respectively (reducing from 55.31 ± 3.89 ng/mL to 39.90 ± 4.72 ng/mL and 37.29 ± 2.45 ng/mL; *p* < 0.001). Similarly, caspase-9 levels decreased by 37.2% and 47.5% (from 6.717 ± 0.65 ng/mL to 4.222 ± 0.57 ng/mL and 3.530 ± 0.47 ng/mL; *p* < 0.001) in comparison to the METH group. These findings highlight the neuroprotective effects of cubebin, as it effectively inhibits the activity of caspase-3 and caspase-9, thereby reducing apoptosis and preserving neuronal viability.

Cubebin was shown to exhibit potent binding affinities toward several proteins related to neurodegenerative diseases. However, the highest degree of preference was established with BDNF (−9.173), followed by CREB (−8.881) and caspase-3 (−7.322). Based on these results, it can be proposed that cubebin may affect the action of these proteins and could, therefore, be useful in the treatment of neurodegenerative diseases.

Ramachandran plots were generated by using the PROCHECK server to analyze the structural compatibility of the protein–ligand complexes. By analyzing these plots, it was found that 4NYX, 1B8M, and 1NME showed favorable conformational thermodynamics, which reflects the structural stability of the structures. In addition, various 2D and 3D visuals of the protein–ligand interactions revealed insightful contributions of molecular interactions in the case of cubebin’s binding affinities. The orientation of 4NYX and cubebin specified the important amino acid residues for the formation of hydrogen bonds, hydrophobic interactions, and other non-covalent associations.

Binding free energies and energy components involving cubebin complexes with 1B8M, 1NME, and 4NYX were determined by MM-GBSA from MD simulations. The result proved that Cubebin_1B8M possesses the highest binding energy (−75.50 ± 5.29 kcal/mol) due to the favorable electrostatic, lipophilic, and VDW interaction energies. Cubebin_1NME bound with moderate energy (−43.76  ±  5.10 kcal/mol), whereby electrostatic and lipophilic interactions were considered to be weaker. Cubebin_4NYX had the lowest binding energy (−38.81 ± 4.71 kcal/mol) because all the energy components had less favorable, lower-energy interactions. Thus, the major forces that contributed to binding included electrostatic and van der Waals forces for all complexes, although lipophilic and covalent interactions differed. While there may have been slightly favorable packing interactions, the effects were minimal on binding energies, and there was a positive solvation energy, indicating that desolvation might be playing an important part in the binding process. These findings are consistent with previous docking studies involving natural compounds targeting neurodegenerative and apoptotic pathways, where similar forces were shown to drive molecular interactions. For instance, coumarin derivatives and polyphenols have demonstrated high affinity for caspase-3 via similar electrostatic and hydrophobic interactions [54,55]. Additionally, natural agents targeting the CREB-BDNF signaling axis have been shown to exert neuroprotective effects through comparable molecular docking and MD simulations [56]. While docking studies specifically involving cubebin are limited, the current findings indicate that it may exhibit mechanistic parallels with other natural compounds targeting similar molecular pathways.

The study faced several constraints, including limited testing animals, a limited timeframe, and complex molecular techniques. The techniques included immunohistochemistry, Western blotting, RT-PCR, and tissue immunohistochemistry. The study was also constrained by the scope of gene protein expression analyses and other genetic modeling approaches. Additionally, in vitro rescue assays, which could have provided further insights into the mechanistic actions and potential cytotoxicity of cubebin at the tested concentrations, were not performed. However, these results support the beneficial effects of cubebin on neurodegenerative diseases for possible therapeutic use. More experimental evidence should be obtained to fully understand the molecular basis of cubebin’s neuroprotective effects and to evaluate its prospects as an effective therapeutic agent.

## 5. Conclusions

This study highlights the promising neuroprotective potential of cubebin against METH-induced cognitive impairments. By alleviating oxidative stress and inflammation and promoting dopamine levels, cubebin effectively mitigated METH-driven learning deficits. These findings suggest that cubebin could serve as a novel therapeutic candidate for managing METH-related neurotoxicity.

Additionally, computational docking studies revealed strong binding affinities of cubebin with key proteins involved in neurodegeneration, including BDNF, CREB, and caspase-3. The observed molecular interactions, as illustrated in both 2D and 3D models, point toward cubebin’s potential role in modulating these targets.

Further research is warranted to explore the precise mechanisms underlying cubebin’s neuroprotective effects and to evaluate its efficacy in clinical settings.

## Figures and Tables

**Table 1:** Comparison between the retrieved protein and standard values in order to validate the protein for the docking study.

Parameters	Details			Standards
Method of Experiment	X-Ray Diffraction			
Protein ID	4NYX	1B8M	1NME	
Mutation	No	No	No	No
Resolution	1.10 Å	2.75 Å	1.60 Å	Near about 3.00 Å
wwPDB Validation	Better	Better	Better	Better
Co-Crystal Ligand	2O4	-	159	-
Ramachandran Plot	96.1%	89.5%	90.2%	>88%

**Table 2:** The active sites’ amino acids.

Protein ID	Active Sites’ Amino Acids
4NYX	ASN1168A, PRO1110A, VAL1115A, LEU1120A, TYR1125A, ARG1173A, VAL1174A, PHE1177A
1B8M	ALA46, ARG53, ARG88, ALA89, ARG98, ALA99, GLN54, GLY99, GLY109, ILE105, PRO43, SER45, LYS46, LYS50, LEU49, GLN51, TYR52, TYR54, TYR86, PHE53, SER85, VAL87, LEU90, ILE98, TRP100, PHE102, THR107, PRO45, LEU52, TYR55, PHE56, SER95, TYR96, VAL97, LEU100, VAL42, VAL44, VAL108, TRP110, TRP112, ILE115, THR117
1NME	TRP206, ARG207, ASN208, SER209, LYS210, TRP214, SER249, PHE250, SER251, PHE252

**Table 3:** Grid parameters.

Sr. No.	Protein ID	Center Coordinates			Size Coordinates		
		x	y	z	x	y	z
1	4NYX	9.51	39.76	12.85	25	25	25
2	1B8M	−0.833	33.139	7.083	25	25	25
3	1NME	42.09	96.34	24.13	25	25	25

**Table 4:** Predicted ADMET properties of cubebin.

Property	Model Name	Predicted Value	Unit
**Absorption**	Water solubility	−4.516	Numeric (log mol/L)
	Caco2 permeability	1.236	Numeric (log Papp in 10^−6^ cm/s)
	Intestinal absorption (human)	97.749	Numeric (% Absorbed)
	Skin permeability	−2.865	Numeric (log Kp)
	P-glycoprotein substrate	Yes	Categorical (Yes/No)
	P-glycoprotein I inhibitor	Yes	Categorical (Yes/No)
	P-glycoprotein II inhibitor	No	Categorical (Yes/No)
**Distribution**	VDss (human)	−0.371	Numeric (log L/kg)
	Fraction unbound (human)	0	Numeric (Fu)
	BBB permeability	−1.111	Numeric (log BB)
	CNS permeability	−3.08	Numeric (log PS)
**Metabolism**	CYP2D6 substrate	No	Categorical (Yes/No)
	CYP3A4 substrate	Yes	Categorical (Yes/No)
	CYP1A2 inhibitor	No	Categorical (Yes/No)
	CYP2C19 inhibitor	Yes	Categorical (Yes/No)
	CYP2C9 inhibitor	No	Categorical (Yes/No)
	CYP2D6 inhibitor	No	Categorical (Yes/No)
	CYP3A4 inhibitor	Yes	Categorical (Yes/No)
**Excretion**	Total clearance	−0.117	Numeric (log ml/min/kg)
	Renal OCT2 substrate	No	Categorical (Yes/No)
**Toxicity**	AMES toxicity	Yes	Categorical (Yes/No)
	Max. tolerated dose (human)	−0.273	Numeric (log mg/kg/day)
	hERG I inhibitor	No	Categorical (Yes/No)
	hERG II inhibitor	Yes	Categorical (Yes/No)
	Oral rat acute toxicity (LD_50_)	2.5	Numeric (mol/kg)
	Oral rat chronic toxicity (LOAEL)	1.584	Numeric (log mg/kg_bw/day)
	Hepatotoxicity	No	Categorical (Yes/No)
	Skin sensitization	No	Categorical (Yes/No)
	*T. pyriformis* toxicity	0.346	Numeric (log ug/L)
	Minnow toxicity	0.159	Numeric (log mM)

ADMET, absorption, distribution, metabolism, excretion, and toxicity; BBB, blood–brain barrier; CNS, central nervous system; CYP, cytochrome; hERG, human ether-à-go-go gene; LD_50_, lethal dose 50; OCT2, organic cation transporter 2; VDss, volume of distribution.

**Table 5:** Docking score ligands with targets CREB, BDNF, and caspase-3 with protein ID 4NYX, 1B8M, and 1NME.

Targets	CREB	BDNF	Caspase-3
**PDB**	4NYX	1B8M	1NME
**Co-crystal ligand**	**2O4**	**-**	**159**
**Cubebin**	−8.881	−9.173	−7.322
**Co-crystal ligand**	−8.645	-	−6.250

**Table 6:** Docking score and intermolecular interactions of ligands with targets CREB, BDNF, and caspase-3 with protein IDs 4NYX, 1B8M, and 1NME, respectively, from PLIP.

PDB	Name of Molecule	Binding Energy	Type of Interaction	Residue ID	Distance
**4NYX**	**Cubebin**	−8.881	Hydrophobic Interactions	LEU1120A	3.58
				LEU1120A	3.76
				ILE1122A	3.92
				TYR1125A	3.34
				VAL1174A	3.81
				VAL1174A	3.49
**1B8M**		−9.173	Hydrophobic Interactions	PHE56B	3.5
				VAL87A	3.91
				ARG88A	3.73
				ARG88A	3.79
				VAL97B	3.28
				ARG98B	3.13
				TRP100A	3.1
				TRP100A	3.48
				PHE102A	3.82
				TRP110B	3.76
			Hydrogen Bonds	PHE57B	2.35
				ARG88A	2.7
				ARG98B	2.65
			Salt Bridges	ARG88A	5.1
**1NME**		−7.322	Hydrophobic Interactions	PHE256B	3.62
			Hydrogen Bonds	TYR204B	3.57
				ARG207B	2.11
			p-Stacking	PHE256B	3.67

**Table 7:** **ΔG_bind_** of the Cubebin_1B8M, Cubebin_1NME, Cubebin_4NYX calculated by MM-GBSA.

Energies (kcal/mol)	Cubebin_1B8M	Cubebin_1NME	Cubebin_4NYX
**ΔG_bind_**	−75.50 ± 5.29	−43.76 ± 5.10	−38.81 ± 4.71
**ΔG_bind_Coulomb**	−18.79 ± 1.84	−8.80 ± 2.84	−10.52 ± 2.62
**ΔG_bind_Covalent**	−0.61 ± 0.86	1.68 ± 1.19	1.11 ± 1.73
**ΔG_bind_H_bond_**	−2.11 ± 0.52	−0.68 ± 0.32	−0.52 ± 0.05
**ΔG_bind_Lipo**	−28.76 ± 1.99	−21.22 ± 2.16	−15.26 ± 1.27
**ΔG_bind_Packing**	−1.08 ± 0.39	−2.23 ± 0.39	−1.55 ± 0.84
**ΔG_bind_SolvGB**	22.45 ± 1.59	14.44 ± 1.75	14.58 ± 1.92
**ΔG_bind_VdW**	−46.61 ± 1.83	−26.95 ± 3.01	−26.66 ± 2.62

## Data Availability

The raw data supporting the conclusions of this article will be made available by the authors on request.

## Supplementary Materials

The following supporting information can be downloaded at https://www.mdpi.com/article/10.3390/medicina61091567/s1: Table S1: Outcome of cubebin on MWM test-Escape latency and time spent in quadrants; Table S2: Outcome of cubebin on neurotransmitter levels-NE, GABA, and DA; Table S3: Outcome of cubebin on antioxidant enzymes-SOD, CAT, and GSH; Table S4: Outcome of cubebin on oxidative stress markers-MDA and NO; Table S5: Outcome of cubebin on neuroinflammatory cytokines, i.e., IL-1β, IL-6, TNF-α, NF-kB, CREB, and BDNF; Table S6: Outcome of cubebin on apoptotic markers-Caspase-3 and Caspase-9.
