# Supplementary material for: Cubebin Attenuates Methamphetamine-Induced Neurotoxicity Through CREB/BDNF/Caspase-3 Signaling: In Vivo and In Silico Study
Source: Medicina (Kaunas). 2025 Aug 31;61(9):1567. doi: 10.3390/medicina61091567 (PMC12472080; doi:10.3390/medicina61091567)
Supplement: Supplementary file 1 [file medicina-61-01567-s001.zip › medicina-3745751-supplementary.pdf]

Table S1: Outcome of cubebin on MWM test-Escape latency and time spent in quadrants.

Groups	Escape latency (s)					Time spent in quadrants (s)
	Day 1	Day 2	Day 3	Day 4	Day 5	
**Control**	24.34±2.03	21.77±1.714	19.45±1.485	17.61±1.77	15.46±2.413	55.33±3.49
**METH**	46.35±4.48	43.16±3.504#	41.76±2.74#	39.43±2.60#	37.06±2.51#	18.24±1.48#
**METH + Cubebin-10**	31.98±2.06	30.12±2.37	31.38±2.835	28.89±1.83∗∗	25.71±2.09∗∗	31.77±2.82∗
**METH + Cubebin-20**	27.57±2.33	26.35±2.861	28.34±2.149	26.2±2.02∗∗∗	22.42±2.52∗∗∗	38.88±4.573∗∗
**Cubebin-20**	24.53±2.10	21.42±1.532	19.67±1.41	17.32±1.65	15.45±1.06	55.25±2.936

Values are expressed in mean ± S.E.M. (n = 6). A one-way ANOVA followed by Tukey's post hoc test, #P < 0.001 vs. normal, ∗P < 0.05, ∗∗P< 0.01, and ∗∗∗P< 0.001 vs. METH.

Table S2: Outcome of cubebin on neurotransmitter levels- NE, GABA, and DA.

Neurotransmitter Levels	Control	METH	METH + Cubebin-10	METH + Cubebin-20	Cubebin-20
**Norepinephrine (ng/mg)**	0.9283±0.16	2.088±0.07#	1.288±0.22∗	1.115±0.17∗∗	0.9083±0.09
**GABA (ng/mg)**	2.290±0.30	0.8433±0.20#	1.902±0.23∗	2.117±0.20∗∗	2.355±0.25
**Dopamine (ng/mg)**	4.013±0.59	9.280±0.76#	6.285±0.64∗	5.482±0.94∗∗	4.257±0.35

GABA-

Values are expressed in mean ± S.E.M. (n = 6). A one-way ANOVA followed by Tukey's post hoc test, #P < 0.001 vs. normal, ∗P < 0.05, and ∗∗P< 0.01 vs. METH.

Table S3: Outcome of cubebin on antioxidant enzymes-SOD, CAT, and GSH.

Antioxidant enzymes	Control	METH	METH + Cubebin-10	METH + Cubebin-20	Cubebin-20
**Superoxide dismutase (U/g)**	13.22±1.33	6.312±0.93#	10.82±0.84∗	12.00±1.03∗∗	13.45±0.60
**Catalase (U/g)**	68.57±3.78	28.73±2.86#	47.38±3.61∗∗	52.53±3.73∗∗∗	69.73±3.85
**GSH (U/g)**	2.190±0.30	0.7000±0.08#	1.563±0.10∗	1.782±0.16∗∗	2.292±0.26

Values are expressed in mean ± S.E.M. (n = 6). A one-way ANOVA followed by Tukey's post hoc test, #P < 0.001 vs. normal, ∗P < 0.05, ∗∗P< 0.01, and ∗∗∗P< 0.001 vs. METH.

Table S4: Outcome of cubebin on oxidative stress markers- MDA and NO.

Oxidative stress markers	Control	METH	METH + Cubebin-10	METH + Cubebin-20	Cubebin-20
**Malonaldehyde (nmol/mg)**	10.47±1.26	21.44±2.44#	14.13±1.80∗	12.16±1.58∗∗	10.27±0.97
**Nitric oxide (nmol/mg)**	0.7550±0.11	1.538±0.16#	1.015±0.12∗	0.8333±0.11∗∗	0.736±0.07

Values are expressed in mean ± S.E.M. (n = 6). A one-way ANOVA followed by Tukey's post hoc test, #P < 0.001 vs. normal, ∗P < 0.05, and ∗∗P< 0.01 vs. METH.

Table S5: Outcome of cubebin on neuroinflammatory cytokines, i.e., IL-1β, IL-6, TNF-α, NF-kB, CREB, and BDNF.

Neuroinflammatory cytokines	Control	METH	METH + Cubebin-10	METH + Cubebin-20	Cubebin-20
Interleukin-1 beta (pg/mL)	44.57±3.61	95.64±4.84 #	77.26±3.76∗	71.13±4.14∗∗	44.85±4.61
Interleukin-6 (pg/mL)	19.07±1.92	42.17±3.11#	30.01±3.49∗	26.48±2.98∗∗	19.62±1.518
Tumor Necrosis Factor-alpha (pg/mL)	60.16±3.477	130.1±3.864#	110.8±5.490∗	102.9±5.704∗∗	59.17±3.221
Nuclear Factor kappa-light-chain-enhancer of activated B cells (pg/mL)	0.5667±0.060	1.525±0.12#	1.048±0.10∗	0.9050±0.07∗∗	0.5533± 0.12
cAMP Response Element-Binding protein (pg/mL)	10.07±0.77	32.76±3.41#	22.25±1.99∗∗	19.03±1.38∗∗∗	10.35±0.90
Brain-Derived Neurotrophic Factor (pg/mL)	210.6±3.83	124.6±5.21#	146.3±3.72∗∗	151.8±4.27∗∗∗	214.0±6.94

Values are expressed in mean ± S.E.M. (n = 6). A one-way ANOVA followed by Tukey's post hoc test, #P < 0.001 vs. normal, ∗P < 0.05, ∗∗P< 0.001 and ∗∗P< 0.0001 vs. METH.

Table S6: Outcome of cubebin on apoptotic markers-Caspase-3 and Caspase-9.

Apoptotic markers	Control	METH	METH + Cubebin-10	METH + Cubebin-20	Cubebin-20
**Caspase 3 (ng/mL)**	21.53±2.22	55.31±3.89#	39.90±4.72∗	37.29±2.45∗∗	21.44±0.94
**Caspase 9 (ng/mL)**	2.575±0.42	6.717±0.65#	4.222±0.57∗	3.530±0.47∗∗	2.743± 0.47

Values are expressed in mean ± S.E.M. (n = 6). A one-way ANOVA followed by Tukey's post hoc test, #P < 0.001 vs. normal, ∗P < 0.05, and ∗∗P< 0.001 vs. METH.
